# Accurate and fast clade assignment via deep learning and frequency chaos game representation

**DOI:** 10.1093/gigascience/giac119

**Published:** 2022-12-28

**Authors:** Jorge Avila Cartes, Santosh Anand, Simone Ciccolella, Paola Bonizzoni, Gianluca Della Vedova

**Affiliations:** Department of Computer Science, Systems and Communications, University of Milano–Bicocca, Milan 20125, Italy; Department of Computer Science, Systems and Communications, University of Milano–Bicocca, Milan 20125, Italy; Department of Computer Science, Systems and Communications, University of Milano–Bicocca, Milan 20125, Italy; Department of Computer Science, Systems and Communications, University of Milano–Bicocca, Milan 20125, Italy; Department of Computer Science, Systems and Communications, University of Milano–Bicocca, Milan 20125, Italy

**Keywords:** chaos game representation, convolutional neural networks, classification of genome sequences, SARS-CoV-2, GISAID clades, *k*-mer frequency, deep learning

## Abstract

**Background:**

Since the beginning of the coronavirus disease 2019 pandemic, there has been an explosion of sequencing of the severe acute respiratory syndrome coronavirus 2 (SARS-CoV-2) virus, making it the most widely sequenced virus in the history. Several databases and tools have been created to keep track of genome sequences and variants of the virus; most notably, the GISAID platform hosts millions of complete genome sequences, and it is continuously expanding every day. A challenging task is the development of fast and accurate tools that are able to distinguish between the different SARS-CoV-2 variants and assign them to a clade.

**Results:**

In this article, we leverage the frequency chaos game representation (FCGR) and convolutional neural networks (CNNs) to develop an original method that learns how to classify genome sequences that we implement into CouGaR-g, a tool for the clade assignment problem on SARS-CoV-2 sequences. On a testing subset of the GISAID, CouGaR-g achieved an $96.29\%$ overall accuracy, while a similar tool, Covidex, obtained a $77,12\%$ overall accuracy. As far as we know, our method is the first using deep learning and FCGR for intraspecies classification. Furthermore, by using some feature importance methods, CouGaR-g allows to identify *k*-mers that match SARS-CoV-2 marker variants.

**Conclusions:**

By combining FCGR and CNNs, we develop a method that achieves a better accuracy than Covidex (which is based on random forest) for clade assignment of SARS-CoV-2 genome sequences, also thanks to our training on a much larger dataset, with comparable running times. Our method implemented in CouGaR-g is able to detect *k*-mers that capture relevant biological information that distinguishes the clades, known as marker variants.

**Availability:**

The trained models can be tested online providing a FASTA file (with 1 or multiple sequences) at https://huggingface.co/spaces/BIASLab/sars-cov-2-classification-fcgr. CouGaR-g is also available at https://github.com/AlgoLab/CouGaR-g under the GPL.

## Introduction

The global coordination in combating the coronavirus disease 2019 (COVID-19) pandemic has led to the sequencing of one of the largest amounts of viral genomic data ever produced. All these data are stored in publicly available archives, such as the European Nucleotide Archive (ENA) and GISAID [1], currently having more than 9.6 million sequenced genomes, classified in *variants, clades*, and *lineages*.

The severe acute respiratory syndrome coronavirus 2 (SARS-CoV-2) virus has evolved since its discovery, and the currently available phylogenies describing its evolutionary history [2] show more than 2,000 different genomes, divided into lineages. Since the phylogeny is fairly stable and the main (existing) *lineages* (i.e., the lines of descent) have been identified, a natural and interesting problem is to quickly find, given a sequence, the *clade* to which it belongs (i.e., a group of descendants sharing a common ancestor) [3]. Fast and efficient solutions to the clade assignment problem would help in tracking current and evolving strains, and it is crucial for the surveillance of the pathogen. This classification problem has been attacked with machine learning approaches [4–6] using the spike protein amino acid sequence to drive the classification step.

In this article, we propose a method for classifying SARS-CoV-2 genome sequences based on chaos game representation (CGR) [7]: a deterministic bidimensional representation of a DNA sequence, also called CGR encoding, that can be easily obtained from the genome sequences. The CGR encoding of a sequence has 2 fundamental properties: it is deterministic (i.e., there is a unique CGR encoding of each sequence) and reversible, and hence the original sequence can be recovered from its representation [8].

A strongly related approach, known as frequency chaos game representation (FCGR) [8, 9], starts from the *k*-mers (the substring of length *k*) of the string we want to represent, resulting in the the notion of *k*th order FCGR [10]. The *kth order FCGR* of a sequence *s* is a 2^*k*^ × 2^*k*^ matrix whose elements are the number of occurrences (i.e., the frequencies) of each *k*-mer in *s*, where each frequency is stored in the specific and distinct position for each *k*-mer. Note that the matrix shape depends on the fact that the sequence *s* is on a 4-symbol alphabet. In essence, the FCGR is an alternative ordering of the histogram for all the *k*-mers (for a fixed integer *k*). Deep learning and FCGR have been used to evaluate the drug resistance for protein sequences of HIV [11]; to identify, for a multiclass classification task, the source organism for a given protein [12] (in this case, the FCGR has been extended to encode sequences in the protein alphabet); and to predict antimicrobial resistance of different drugs in *Escherichia coli* [13]. The FCGR has also been used for unsupervised clustering of DNA sequences of several species [14] by using dense neural networks, where the input of these networks must be a 1-dimensional vector. In this case, the 2-dimensional FCGR representation of the sequences must be flattened and cannot be fully exploited. For an extensive review on CGR and its applications in bioinformatics, we refer the reader to [15].

Subtyping SARS-CoV-2 sequences has been addressed in the literature with bioinformatics pipelines that require the alignment to a reference genome  [2, 16] and also with machine learning approaches aiming to skip the alignment step  [17]. Furthermore, an early approach to construct phylogenetics trees within SARS-CoV-2 strains and closely related species was proposed in [18] using FCGR as embedding for a hierarchical agglomerative clustering. In a similar fashion, FCGR was explored along with other techniques as embedding for the identification of homologies between different known and emerging viruses in [19]. Convolutional neural networks (CNNs) [20] showed outstanding results in the well-known Imagenet classification problem [21]. To the best of our knowledge, only 2 works have used CNNs and FCGR for the classification of DNA sequences. In [22], a simplification of the network reported in was used to classify different taxonomic categories with a dataset of 3,000 sequences (1,200–1,400 long). A comparison with support vector machines (SVMs) showed that CNNs improve over SVM when using a fragment (500 bp) of the sequences. In [23], a CNN was proposed for the classification of a dataset of ≈660 sequences from 11 phylogenetic families reporting a test accuracy of $87\%$.

In this article, we leverage the FCGR representation of genomic sequences and CNN power to perform intraspecies classification of viral DNA genome sequences, using SARS-CoV-2 as our case of study and GISAID clades as our labels. Observe that in this problem, the CNN classifies a dataset that is at least 2 orders of magnitude larger than the one considered in the abovementioned studies. Another work that has tackled the clade assignment problem is Covidex [24], a web app tool based on random forest and *k*-mer frequencies: to the best of our knowledge, this is the most recent work facing our problem. Notice that almost the entire phylogenetics literature deals with interspecies classification, where the distance between possible cluster centroids is larger, and hence the classification problem is easier. We propose using a residual neural network [25] (ResNet50) for the classification of DNA sequences into 11 GISAID clades, using a dataset of 2 orders of magnitude larger (153,000 sequences for training) than those analyzed in the above-cited works (about 3,000 sequences in [22]).

Classification metrics (accuracy, Matthews correlation coefficient [26], precision, recall, and F1-score) and analysis of the separability of the embeddings generated by the classification layer (silhouette coefficient [27], Calinski–Harabasz score [28], and generalized discrimination value [GDV] [29]) are analyzed for each model. Using the fact that each feature in the FCGR is uniquely related to a *k*-mer, we aim to analyze if the most relevant *k*-mers identified by feature importance methods (saliency maps [30] and Shapley additive explanations [SHAP] values [31]) are related to mutations defining each clade.

We trained 4 models, one for each value of *k* ∈ {6, 7, 8}. All models performed very similarly, with *k* = 8 being the best one, achieving an overall accuracy of $96.22\%$ in the test set and the best classification metrics (0.948 for silhouette coefficient, 174,736.1 for Calinski–Harabasz, and −0.718 for GDV). Three clades (O, GR, and GRY) reported the lowest F1-score for all the trained models. Since GR is a close ancestor of GRY and these 2 clades share many mutations, they are confused with each other. For clade O, mispredictions are among most of the clades.

Using the 20 most relevant *k*-mers identified by saliency maps, we were able to achieve a similar performance to our CNN models using SVM for *k* ∈ {6, 7, 8}. Finally, to access the performance of our models with respect to (w.r.t.) other approaches, we compare our results with Covidex [24], the only recent tool that we found in the literature solving the clade assignment problem. Our results show that our models outperform Covidex in all clades and reported metrics (accuracy, precision, recall, and F1-score).

## Background

The CGR for encoding DNA/RNA sequences is formally defined as follows:

### Definition 1 (CGR)

*Let s* = *s*_1_…*s_n_* ∈ {*A, C, G, T*}* *be a sequence. Then the CGR encoding of the sequence s is the bidimensional representation of the ordered pair* (*x_n_*, *y_n_*), *which is defined iteratively as*  

(1) \begin{eqnarray*} (x_i,y_i) = \frac{1}{2}\Big ((x_{i-1},y_{i-1}) + g(s_i)\Big )\text{, if }i\ge 1
\end{eqnarray*}

*where* (*x*_0_, *y*_0_) = (0, 0) *and*  

(2) \begin{eqnarray*}
g(s_i) = \left\lbrace \begin{array}{ll}(1,1) & s_i = A \\ (-1,1) & s_i = C \\ (-1,-1) & s_i = G \\ (1,-1) & s_i = T \end{array} \right. \end{eqnarray*}

Note that each point (*x_i_*, *y_i_*) obtained with the above encoding represents the *i*-long prefix of the sequence *s*. Also, all the CGR encodings are points inside the square with vertices given by the values of the function *g*. In particular, the encoding of all prefixes that shares the last character will be placed in the same quadrant; all prefixes that share the 2 last characters will be placed in the same sub-quadrant, and so on. This property results in a fractal structure of the representation.

Missing bases can be problematic to encode, since the *g*( · ) function is not defined in that case; we used the notion of frequency matrix CGR [8, 9], which has the added benefit of allowing us to manage *k*-mers instead of strings of arbitrary length.

### Definition 2 (frequency matrix of CGR)

*Let s* = *s*_1_…*s_n_* ∈ {*A, C, G, T, N*}* *be a sequence, and let k be an integer. Then the frequency matrix of CGR, in short FCGR, of the sequence s is a* 2^*k*^ × 2^*k*^  *bi-dimensional matrix*$F=(a_{i,j}),1\le i,j\le 2^k, i,j \in \mathbb {N}$. *For each k-mer b* ∈ {*A, C, G, T*}^*k*^, *we have an element a_i, j_in the matrix F that is equal to the number of occurrences of b as a substring of s. Moreover, the position* (*i, j*) *of such element is computed as follows:*  

\begin{eqnarray*}
i =& 2^k - \big \lceil 2^{k-1}(x+1)\big \rceil + 1 \\ j =& \lceil 2^{k-1}(y+1) \rceil
\end{eqnarray*}

*where* (*x, y*) *is the CGR encoding for the k*-mer *b*.

Note that the FCGR is defined for a DNA sequence with unknown nucleotide, denoted by *N*—where *k*-mers with an *N* are simply excluded in the counting process—while the *CGR* encoding is well defined only when all nucleotides are known. To explicitly mention the dimension of the FCGR, we will refer to this as the *k*th-order FCGR.

### Classification of viral sequences of DNA

We are given a phylogeny over the possible viral strains, partitioned into classes: each class *c* of such partition $\mathcal {C}$ is a *clade* of the tree. More precisely, a clade is a group of related organisms descended from a common ancestor [3]; in other words, a clade is a subtree of a phylogeny that consists of an ancestral lineage and all its descendants.

Given a genome sequence, which is a string *s* ∈ {*A, C, G, T, N*}*, we determine the original clade in $\mathcal {C}$ from which the genome sequence is originated; however, the genome sequence *s* might not have been previously observed. In any case, the sequence will be assigned to a putative clade. To solve this problem, we propose a supervised learning model based on CNNs, using FCGR as inputs.

## Data description

The dataset for this experiment was downloaded from GISAID. By the time of our access to GISAID (https://www.gisaid.org/; 4 April 2022), there were around 10 million sequences.

In order to undersample the available data, we first dropped all the rows in the metadata without information in the columns Virus name, Collection Date, Submission Date, clade, Host, and Is complete?, and then we built a fasta_id identifier from the metadata as a concatenation of the columns Virus name, Collection Date, and Submission Date.

For each clade, we randomly selected 20,000 sequences considering only those rows where the Host column has value “Human”—clades L, V, and S have fewer than 20,000 sequences available, and in these cases, all sequences have been selected.

As a result of the above procedure, we obtained 191,456 sequences among the 11 GISAID clades (S, L, G, V, GR, GH, GV, GK, GRY, O, and GRA) over the 12 available, and we excluded the clade GKA from our study since there were only 81 sequences reported in the metadata. The undersampled dataset was randomly split into train, validation, and test sets in a 80:10:10 proportion, preserving the same proportion of clades (labels) in each set. The distribution of the clades over the datasets is given in Table 1.

**Table 1:** Distribution of the number of sequences selected for train, validation, and test sets by each clade. The final dataset for the 11 clades was split in a 80:10:10 proportion for train, validation, and test sets.

Clade	Train	Val	Test	Total	Available
S	14,298	1,788	1,788	17,874	17,874
L	5,154	644	644	6,442	6,442
G	15,999	2,000	2,000	20,000	408,552
V	5,713	714	714	7,141	7,141
GR	16,000	2,000	2,000	20,000	625,662
GH	16,000	2,000	2,000	20,000	547,792
GV	16,000	2,000	2,000	20,000	182,248
GK	16,000	2,000	2,000	20,000	4,170,758
GRY	16,000	2,000	2,000	20,000	944,876
O	16,000	2,000	2,000	20,000	55,400
GRA	16,000	2,000	2,000	20,000	2,833,863
Total	153,164	19,146	19,146	191,456	9,800,608

## Analyses

In this section, we present the experimental setup, the dataset used to train and test each model, and clustering and classification metrics. We train 1 model for each *k* ∈ {6, 7, 8}, and we complement the study of the accuracy of each model (compared against Covidex [24]) with an analysis of the most relevant *k*-mers for the classification of each clade using saliency maps and SHAP.

For this experiment, we choose *k* ∈ {6, 7, 8} and sequences from 11 GISAID clades: S, L, G, V, GR, GH, GV, GK, GRY, O, and GRA.

### Experimental setup

All experiments are conducted using a Intel Core i5-10400 CPU @ 2.90 GHz, x86_64, 32 GB RAM, and a graphic card NVIDIA GeForce RTX 3060. The implementation is done in Python 3.10.5. Tensorflow 2.10.0 [32] was used for training the CNN and scikit-learn 1.1.12 [33] to compute classification metrics and clustering evaluation (except for GDV that was implemented). All code is available online for reproducibility (https://github.com/AlgoLab/CouGaR-g).

### Model training

Each model was set to be trained for 50 epochs with a batch size of 32 using an Adam optimizer [34] with a learning rate of 0.001 (the default parameters in keras). The validation loss was monitored after each epoch to save the best-trained weights, reducing the learning rate with a patience of 8 epochs and a factor of 0.1, and by an early stopping in case the metrics did not improve after 12 epochs.

**Figure 1:**
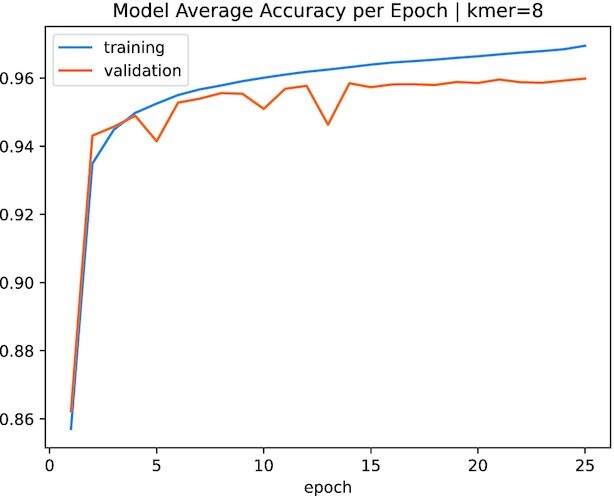
Average accuracy in the training and validation sets for our model with *k* = 8. The best model (final weights) is set as the one with the lowest validation loss, achieved at epochs 24 ± 5 for *k* = 8 (from a repeated 5-fold cross-validation process). All models were trained for 50 epochs using an early stopping of 12 epochs based on the validation loss (hence, not all of them ran for 50 epochs).

We show the accuracy (average of the repeated 5-fold cross-validation) of the train and validation sets for *k* = 8 in Fig. 1. For *k* = 6 and *k* = 7, the training is more unstable for the first epochs, but it behaves similar to *k* = 8 in the later epochs (i.e., training and validation metrics are similar).

The architecture used in this experiment is the same for all *k* (ResNet50 [25]); we only changed the input size. Originally, this architecture was designed for inputs of size (224 × 224 × 3), which led us to the assumption that this architecture could be more suitable for *k* = 8. Notice that our sequences are ≈29,000 bp long, which means that our input FCGR for *k* = 8 is very sparse, since from an *n*-long sequence, we can count *n* − *k* + 1 *k*-mers. This means that (in the case where all *k*-mers are different) we have at most 29,000 *k*-mers, and at least $55\%$ of the elements of the FCGR are 0 for *k* = 8. In Table 2, a comparison of the number of features for each *k* and the training time per epoch in our experiments are detailed.

**Table 2:** For each *k*, the dimension of the FCGR, its number of features (4^*k*^), the amount of memory required to store the selected dataset of 191,456 sequences as FCGR, and the average training time per epoch are reported. The number of features and the space increase exponentially with respect to *k*.

*k*-mer	Dimensions	Features	Size (GiB)	Time per epoch (min)
6	(64,64)	4,096	6.6	4:05
7	(128,128)	16,384	24.1	8:21
8	(256,256)	65,536	94.2	24:50

### Classification results

After each model is trained, Accuracy and Matthews correlation coefficient are reported as global metrics (see Table 3). The precision and recall for the test set are computed for each clade using the best-trained weights (lowest loss in the validation set), achieved at epochs 24 ± 5, 27 ± 8, and 20 ± 3 for *k* = 6, *k* = 7, and *k* = 8, respectively. In our case, we assign each sequence to the clade with the highest score. Precision, recall, and F1-score are shown in Table 4.

**Table 3:** Accuracy and Matthews correlation coefficient (MCC) in the set for each of our models. Each metric (μ ± σ) is reported by its average (μ) and standard deviation (σ) from a repeated 5-fold cross-validation process. For both accuracy and MCC, the model increases with the value of *k*. Going from *k*= 6 to *k*= 8 increases accuracy in 0.84% and MCC in 0.94%. The highest value of each metric is highlighted in bold.

*k*-mer	Accuracy	MCC
6	0.953714 ± 0.001589	0.948792 ± 0.001813
7	0.959856 ± 0.001740	0.955566 ± 0.001910
8	**0.962175** ± **0.002829**	**0.958211** ± **0.003141**

**Table 4:** Precision, recall, and F1-score. Each of our models is represented by length of the the *k*-mers used to generate the FCGR. Two clades, GR and GRY, present deviations greater than 1% in their precision and recall for all values of *k*. The highest F1-score for each clade and *k* is highlighted in bold. Each metric (μ ± σ) is reported by its average (μ) and standard deviation (σ) from a repeated 5-fold cross-validation process.

*k*-mer		6			7			8	
Clade	Precision	Recall	F1-score	Precision	Recall	F1-score	Precision	Recall	F1-score
S	99.4 ± 0.2	99.6 ± 0.2	99.5 ± 0.1	99.7 ± 0.1	99.6 ± 0.1	99.7 ± 0.1	99.8 ± 0.2	99.7 ± 0.3	**99.8** ± **0.1**
L	98.3 ± 0.4	97.7 ± 0.6	98.0 ± 0.3	98.7 ± 0.4	99.0 ± 0.2	**98.9** ± **0.2**	98.0 ± 0.4	99.5 ± 0.3	98.7 ± 0.2
G	95.8 ± 0.7	94.7 ± 0.8	95.2 ± 0.4	97.1 ± 0.4	95.1 ± 0.4	96.1 ± 0.3	97.3 ± 0.8	95.9 ± 0.7	**96.6** ± **0.1**
V	99.1 ± 0.4	99.2 ± 0.4	99.1 ± 0.2	99.5 ± 0.5	99.4 ± 0.3	99.4 ± 0.3	99.6 ± 0.5	99.6 ± 0.2	**99.6** ± **0.3**
GR	91.7 ± 2.0	85.9 ± 1.7	88.7 ± 0.4	92.4 ± 1.3	87.5 ± 1.4	89.8 ± 0.2	93.9 ± 2.3	86.6 ± 1.7	**90.1** ± **0.7**
GH	98.6 ± 0.3	99.5 ± 0.1	99.0 ± 0.2	98.9 ± 0.2	99.7 ± 0.1	**99.3** ± **0.1**	98.8 ± 0.2	99.8 ± 0.1	**99.3** ± **0.1**
GV	99.5 ± 0.3	99.6 ± 0.2	99.5 ± 0.1	99.7 ± 0.1	99.6 ± 0.1	99.7 ± 0.1	99.6 ± 0.1	99.8 ± 0.1	**99.7** ± **0.0**
GK	91.8 ± 0.4	97.6 ± 0.5	94.6 ± 0.2	92.2 ± 0.5	97.7 ± 0.2	94.9 ± 0.4	92.7 ± 0.2	98.1 ± 0.8	**95.3** ± **0.4**
GRY	86.4 ± 1.3	93.2 ± 2.4	89.7 ± 0.6	87.8 ± 1.1	93.9 ± 1.4	90.7 ± 0.3	87.1 ± 1.5	95.5 ± 2.0	**91.0** ± **0.7**
O	94.3 ± 0.8	86.8 ± 0.2	90.4 ± 0.4	95.0 ± 0.6	89.2 ± 0.8	92.1 ± 0.6	96.7 ± 0.4	88.8 ± 1.2	**92.6** ± **0.7**
GRA	99.7 ± 0.1	99.8 ± 0.1	99.8 ± 0.1	99.9 ± 0.1	99.7 ± 0.1	**99.8** ± **0.0**	99.8 ± 0.1	99.8 ± 0.1	99.8 ± 0.1

**Figure 2:**
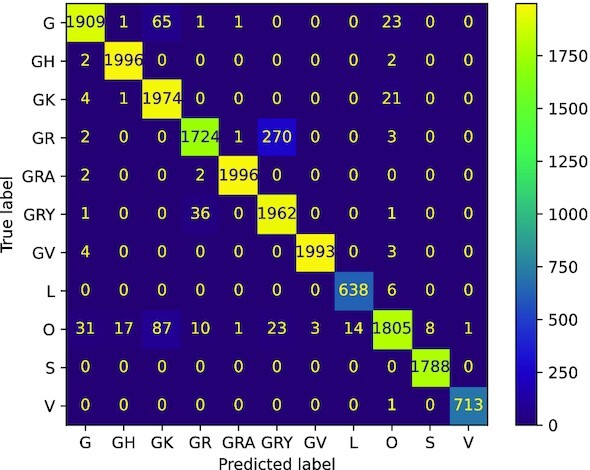
Confusion matrix for the test set for one of the trained models with *k* = 8 (from a repeated 5-fold cross-validation process). All the models are able to correctly classify more than $98\%$ of the sequences for all clades except for G, GR, GRY, and O. Most of the incorrectly classified sequences of GR and GRY are confused between them, which makes sense since they are evolutionary related. For the G clade, the incorrectly classified sequences are shared between clades GK and O. For the clade O, the incorrectly classified sequences are predominantly assigned to clades G, GH, GK, and GRY.

Precision and recall are very similar among all the trained models, with small improvements when *k* increases; 5 out of 11 clades have an F1-score greater than $99\%$ in our best model (*k* = 8). Most notable differences in the performance can be seen in clades GR and GRY, which present the lowest (and under $90\%$) reported recall and precision in each model, respectively. Moreover, from the confusion matrices (see Fig. 2), we can see that misclassified sequences that belong to clades GR and GRY are confused between them, and this can be explained since clade GRY originates from clade GR. For the other clades, most of the misclassified sequences are predicted as (or belong to) clade G, which is the former one. Clade O exhibits the second lowest recall, where the misclassified sequences are assigned predominantly to clades G, GH, GK, and GRY.

### Comparison with the literature

We compare our results against Covidex [24], a tool that classifies SARS-CoV-2 sequences into 3 nomenclatures: GISAID, Nexstrain, and Pango lineages. Using a different model for each task, all based on random forest and 6-mers as input, the reported accuracies are $97,77\%$, $99,52\%$, and $96,56\%$ for GISAID, Nextstrain, and Pango models, respectively. Covidex also trained the models using 7-mers but claimed that it only produced slightly better results in terms of accuracy but with more than doubling the computation time  [24].

The input for Covidex is a vector with the normalized counting of the frequencies for all 4^*k*^  *k*-mers. Our input, the FCGR, also considers all *k*-mers but in a bidimensional matrix. The main difference between both approaches is the model behind it; while Covidex uses random forest to perform the classification, we take advantage of the CNNs and use a 2-dimensional input, the FCGR. Notice that using the FCGR with any other classical machine learning method implies converting the FCGR into a vector and, hence, the loss of the 2-dimensional structure.

Since our model is trained using GISAID clades, we only compare to those results. Covidex used 10 clades: S, L, G, V, GR, GH, GV, GK, GRY, and O. In our case, we included GRA since there were enough available sequences by the time of our experiments, but this is not considered in the comparison.

For Covidex, the model for the GISAID nomenclature was trained with 66,126 sequences and tested on 13,230. Since Covidex is made available as an user app for any SARS-CoV-2 sequence, we used the app over our test dataset to compare the results. We tested Covidex on our test dataset of 17,146 sequences (excluding the 2,000 sequences from GRA clade). We achieved an accuracy of $77,12\%$, more than $18\%$ lower than all our trained models and $20,65\%$ lower than their reported accuracy. The reported precision, recall, and F1-score, as well as the test results over our selected dataset, can be seen in Table 5. We found that the reported F1-score of Covidex is quite distant from the one we obtained in our test dataset for clades L ($-8.4\%$), G ($-15.8\%$), GR ($-42.4\%$), GK ($-10.9\%$), GRY ($-19.3\%$), and O ($-28.8\%$), while for clades S ($-0.8\%$), V ($-2.9\%$), GH ($-2.5\%$), and GV ($-0.9\%$), we can observe a decrement on the reported F1-score ranging from $0.8\%$ to $2.9\%$. Our models (see Table 4) exhibit better performance than Covidex in all clades and metrics on our test set, with similar results only on clades S and GV. We did not perform an extensive comparison of the running times since both tools classify a genome sequence in less than a second (on *k* = 8, our tool took 0.15 seconds on average).

**Table 5:** Precision, recall, and F1-score for Covidex. The report part is taken from the supplementary material of [24]. The test part has the precision, recall, and F1-score obtained by Covidex on our test set, restricted to the 10 clades (17,146 sequences) analyzed in [24]. We found significant differences between Covidex and our trained models in the test metrics (see Table 4). In particular, the most notorious differences with respect to F1-score, ranging from 8.4% to 42.4%, are found for clades L ($-8.4\%$), G ($-15.8\%$), GR ($-42.4\%$), GK ($-10.9\%$), GRY ($-19.3\%$), and O ($-28.8\%$), while for clades S ($-0.8\%$), V ($-2.9\%$), GH ($-2.5\%$), and GV ($-0.9\%$), we can observe a decrement in the reported F1-score ranging from $0.8\%$–$2.9\%$. Metrics in bold in the test part are those that *did not decrease* more than 3% with respect to the reported metrics.

		Report			Test	
Clade	Prec.	Rec.	F1-score	Prec.	Rec.	F1-score
S	0.998	1	0.999	**0.988**	**0.995**	**0.991**
L	0.997	1	0.999	0.859	**0.979**	0.915
G	0.993	0.984	0.989	0.811	0.852	0.831
V	1	1	1	0.958	**0.985**	**0.971**
GR	0.945	0.915	0.930	0.379	0.760	0.506
GH	0.995	0.999	0.997	0.957	**0.987**	**0.972**
GV	0.996	0.999	0.997	**0.980**	**0.995**	**0.988**
GK	0.977	0.995	0.986	0.925	0.833	0.877
GRY	0.920	0.961	0.940	0.732	0.763	0.747
O	0.994	0.959	0.976	0.722	0.658	0.688

### Clustering results

We evaluate the embeddings of the last layer of each trained model using the silhouette coefficient, Calinski–Harabasz score, and GDV. These results are shown in Table 6. We can observe that the model for *k* = 9 is the best one among all metrics, but all trained models exhibit a very similar separability based on silhouette and GDV.

**Table 6:** Clustering metrics for our trained models. Each metric is computed using the output of each model and the predicted clade (i.e., the clade that achieves the highest score by our model) in the test set. Each model is represented by the length of the *k*-mers used to generate the FCGR. For the silhouette score, the closest to 1 the better. For the Calinski–Harabasz score, larger values are better. For the GDV score, the closest to −1, the better. All models exhibit comparable separability of the clusters. Each metric (μ ± σ) is reported by the average (μ) and standard deviation (σ) in the form of a repeated 5-fold cross-validation process.

*k*-mer	Silhouette	Calinski–Harabasz	GDV
6	0.939 ± 0.007	145,879.214 ± 18,132.428	−0.712 ± 0.003
7	0.948 ± 0.003	163,926.767 ± 8,638.391	−0.717 ± 0.002
8	0.948 ± 0.006	174,736.086 ± 22,554.904	−0.718 ± 0.003

### Relevant *k*-mers for the classification of each clade

The purpose of this experiment is to study if a set of the most relevant *k*-mers (based on feature importance methods) are informative enough to a SVM to perform similarly to the trained CNNs (that uses FCGR as input and hence all the 4^*k*^ possible *k*-mers).

Using saliency maps and SHAP values, we can evaluate the contribution of each element of a FCGR in the classification, for each model. From each of these feature attribution methods, we can obtain an ordered list of all *k*-mers. For each clade, we use the centroid FCGR of all correctly classified sequences in the test set, and then we use each centroid FCGR to identify the most relevant *k*-mers for each clade and then train a SVM using the *N* most relevant *k*-mers (for different values of *N* ∈ {1, 2, 3, 4, 5, 10, 15, 20, 25, 30, 35, 40, 45, 50}) and their respective frequencies as input.

The same training and test sets used for the CNNs were used for the SVM. The results of the accuracy in the test set for the different values of *N* are shown in Figs. 5 and  6. We can observe that *k*-mers identified by saliency maps are more informative than those identified by SHAP values, since for *N* = 20, we obtain similar accuracy in the test set for *k* = 6, 7, 8 compared to CNN (96–$97\%$), while in the case of SHAP values, this accuracy is only achieved by *k* = 7 with *N* = 35. Notice that using *N* = 20, we are considering a small number of all possible *k*-mers ($0.49\%$ for *k* = 6, $0.12\%$ for *k* = 7, and $0.03\%$ for *k* = 8).

### Matching relevant *k*-mers to mutations

Using the reference genome employed by GISAID (EPI_ISL_402124) (https://www.gisaid.org/resources/hcov-19-reference-sequence/) and the list of marker variants (https://www.gisaid.org/resources/statements-clarifications/clade-and-lineage-nomenclature-aids-in-genomic-epidemiology-of-active-hcov-19-viruses/) for each GISAID clade with respect to this reference, we evaluated how many *k*-mers among the 50 chosen ones by saliency maps and SHAP values actually matched any of the reported marker variants. A summary is shown in Table 7.

**Table 7:** Summary of matches between the 50 most relevant *k*-mers (from saliency maps and SHAP values) and the list of marker variants reported by GISAID for each clade. The *k*-mers obtained by saliency maps are able to match several mutations and the matches decrease when *k* increases, but the ones from SHAP values only reported 3 matches, for *k* = 6.

*k*-mer	Saliency maps	SHAP values
6	46	3
7	51	0
8	11	0

**Figure 3:**
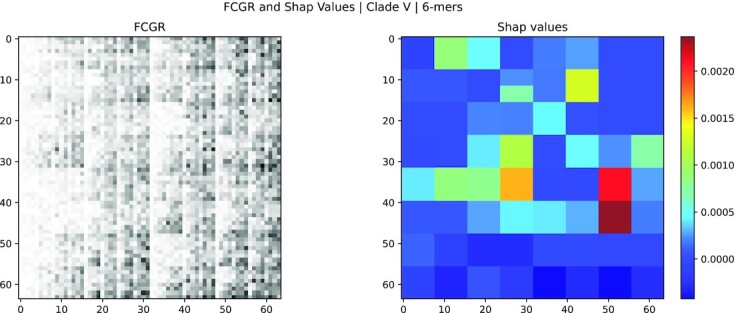
FCGR image (left) and SHAP values (right) of the centroid FCGR for the clade V (*k* = 6). The FCGR image is obtained rescaling the frequencies in the FCGR to a gray-scale range of 8 bits ([0, 255]); an inversion of colors is performed to visualize higher values as black squares and lower values as white. SHAP values represent the importance of the features in the FCGR; the higher the value (red), the more important is the feature. Each feature (pixel) in the FCGR corresponds to a *k*-mer.

**Figure 4:**
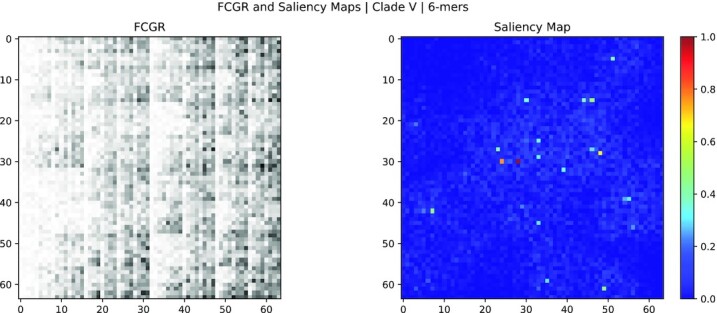
FCGR image (left) and saliency map (right) of the centroid FCGR for the clade V (*k* = 6). The FCGR image is obtained rescaling the frequencies in the FCGR to a gray-scale range of 8 bits ([0, 255]); an inversion of colors is performed to visualize higher values as black squares and lower values as white. Saliency maps represent the importance of the features in the FCGR; the higher the value (red), the more important is the feature. Each feature (pixel) in the FCGR and saliency map corresponds to a *k*-mer.

The results show that the most relevant *k*-mers selected using saliency maps match several of the reported marker variants (46 matches for *k* = 6, 51 for *k* = 7, and 11 for *k* = 8). On the other hand, the ones chosen by SHAP values barely match with the mutation (3 for *k* = 6), suggesting that saliency maps could provide a richer explainability of the model from a biological perspective. Examples of SHAP values and Saliency Maps applied to a random sample from clade V are shown in Figs. 3 and 4, respectively.

**Figure 5:**
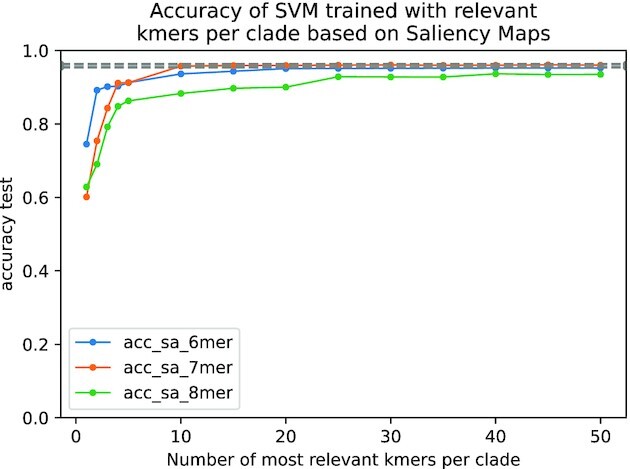
Accuracy of test set for SVM trained models using only the most *N* relevant *k*-mers for each clade (*N* ∈ {1, 2, 3, 4, 5, 10, 15, 20, 25, 30, 35, 40, 45, 50}). The relevant *k*-mers are selected using saliency maps on the centroid of the correctly classified FCGR for each clade and model. The same train and test datasets used for the trained CNNs are used for the SVM. The SVM trained with 20 most relevant *k*-mers identified by the saliency map, for *k* ∈ {6, 7} achieves an accuracy in the test set ($\approx 96\%$) that is in the range of the minimum and maximum accuracies (see Table 3) obtained by our trained CNNs (the gray dashed band represents the minimum and maximum accuracy for the trained CNNs).

**Figure 6:**
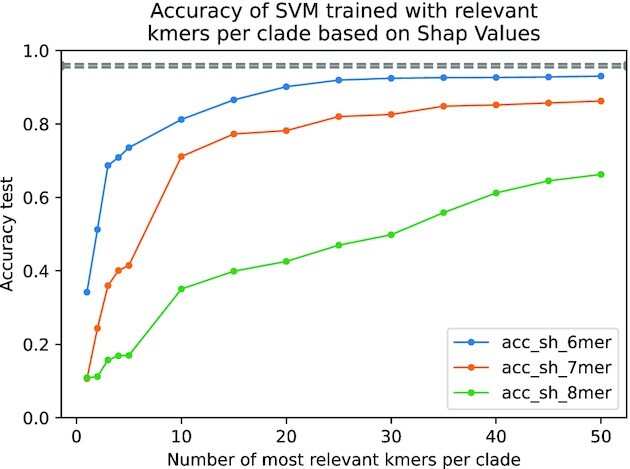
Accuracy of test set for SVM trained model using only the most *N* relevant *k*-mers for each clade (*N* ∈ {1, 2, 3, 4, 5, 10, 15, 20, 25, 30, 35, 40, 45, 50}). The relevant *k*-mers are selected using SHAP values on the centroid of the correctly classified FCGR for each clade and model. The same train and test datasets used for the trained CNNs are used for the SVM. The SVM, trained with the 30 most relevant (or more) 6-mers identified by SHAP values, achieves the closest accuracy ($92,44\%$) to the ones obtained by our trained models (see Table 3). When *k* increases, the accuracy always decreases (for the same number of relevant *k*-mers), which can be explained since when *k* increases, the total number of possible *k*-mers increases exponentially.

## Discussion

In this work, we have shown that FCGR can be used to classify DNA sequences. Most notably, we have used FCGR to assign SARS-CoV-2 genome sequences to its GISAID strain by running a CNN on 191,456 genome sequences ($80\%$ training set, $10\%$ validation set, and $10\%$ test set). In particular, the eighth-order FCGR achieved a test accuracy of $96.22\%$.Most misclassified sequences are shared between 2 strongly related strains, GR and GRY (GR is a close ancestor of GRY).

We decided to exclude transfer learning from our experiments after trying this approach without success on 8-mers. For this trial, we used pretrained weights from the Imagenet dataset using ResNet50 architecture, where the backbone weights were frozen, and 3 dense layers were included at the top of it for the classification.

We have assessed the influence of the length *k* of the substrings (*k*-mers) used to build the FCGR, showing that values between 6 and 8 lead to very similar results, with less than 1% of difference in both accuracy and MCC on the same test set. However, when increasing the value of *k*, the training time for the model and the memory required to save the FCGRs increases exponentially. For *k* = 6, each epoch required 4:05 minutes and 6.6 GB of memory, while for *k* = 8, it required 24:50 hours and 94.2 GB. However, FCGRs show fractal structures; this suggests that we might couple increasing *k* with using only a portion of the FCGR.

We compare our results with Covidex, a random forest–based tool that classifies sequences on GISAID clades based on *k*-mer frequencies. Under the same test set, our results show that our models outperform Covidex in all clades and reported metrics (accuracy, precision, recall, and F1-score). Moreover, we found that the reported precision, recall, and F1-score of Covidex are quite different for all clades except S and GV in our test set, exhibiting a decrease in the F1-score metric up to 42.4%.

We have used saliency maps and SHAP to identify relevant *k*-mers, looking for matches with the marker variants reported for each strain. Using the *k*-mers obtained by saliency maps, we found 46, 51, and 11 matches for *k* = 6, 7, and 8, respectively. While, for the *k*-mers identified by SHAP, only 3 matches were found for *k* = 6. A possible direction for future work is to explore other existing methods (e.g., Lime [35], GradCAM [36], DeepLIFT [37]) that might be suitable in explaining the decisions of the model.

Classifying genome sequences by introducing the assembly bias includes more factors to take care of, since any classification depends on the specific assembly pipeline that has been used. To lessen this possible problem, we should study a related problem, where we classify read samples instead of fully assembled genomes. This new problem is more complex, since different regions of the viral genome can have different coverage—hence impacting the frequencies—and reads need to be cleaned from both errors and contamination artifacts (the latter might be attacked with specialized tools like KMC3 [38]).

We did not perform an extensive comparison of the running times since both tools classify a genome sequence in less than a second.

## Potential implications

This article shows how to couple FCGR with a deep neural network that is especially suited to represent images, such as a CNN, to predict clade assignment. Since FCGR is a simple and intuitive representation of a set of *k*-mers, we expect this combination to find applications in several other problems that are currently attacked with approaches based on *k*-mers.

## Methods

We use the *k*th-order FCGR representation for each sequence. In order to obtain this representation, we need to count the *k*-mers in each sequence and to put those frequencies in the FCGR based on the CGR encoding.

Before feeding the FCGR to the model, we rescale its elements to values between 0 and 1 for stability of the learning process. To do so, we divide each FCGR element-wise by the maximum value in the FCGR. It is worth mentioning that other preprocessing steps were taken into consideration but ultimately excluded because they were empirically worse.

Due to the huge amount of data available for most of the clades, we undersampled at most 20,000 sequences per clade to perform our experiments; nevertheless, for some clades, only a portion was available (see table representativity). In order to overcome the unbalance in the undersampled dataset, we decided to use a weighted binary cross-entropy loss function (instead of oversampling the underrepresented classes), where the cost associated with a class *c* is inversely proportional to its representativity in the training set.

### Model architecture

We choose a residual neural network, ResNet50 [39], as our CNN, adapted for *k*th-order FCGR, that is, with input size equal to (2^*k*^ × 2^*k*^ × 1) and output size equal to the number of clades: $|\mathcal {C}|$, with softmax activation function in the last layer and categorical cross-entropy as loss function, since we want to assign only 1 clade to each DNA sequence.

### Model evaluation

To assess the performance of our trained model, we perform a classification evaluation of the predictions and also a clustering evaluation for the embeddings in order to evaluate the class separability. The reported metrics are based on a repeated 5-fold cross-validation.

#### Classification metrics

We report global (accuracy and Matthews correlation coefficient) and class specific metrics (precision, recall, and F1-score) for the trained models.

#### Class-specific metrics

Given a clade *c*, the correct predictions of the model can be compared to all the sequences with ground truth *c* (recall) and to all the sequences predicted by the model into the clade *c* (precision).

Formally, given a clade *c*, the positive class *P* consists of the set of genome sequences that are assigned to *c*, while all other genome sequences are the negative class *N*. Consequently, the true positives consist of the sequences that originate from the clade *c* and have been assigned to *c*, the false positives consist of the sequences that do not originate from the clade *c* and have been assigned to *c*, and the false negatives consist of the sequences that originate from the clade *c* and have not been assigned to *c*. The precision and recall are computed as follows:

(3) \begin{eqnarray*}
precision = \dfrac{TP}{TP+FP}, recall = \dfrac{TP}{TP+FN}
\end{eqnarray*}

We also report the F1-score, defined as

(4) \begin{eqnarray*}
f1-score = 2\dfrac{precision \times recall}{precision + recall}
\end{eqnarray*}

#### Global model metrics

Given a classification problem on *S* samples and *N* classes, the corresponding confusion matrix *C* = (*c_ij_*), *i, j* ∈ [1, *N*] is a square matrix where each entry *c_i, j_* is the number of elements that belong to the true class *i* and were classified in the class *j*, and the sum of the entries in *C* is exactly *S*.

- The **accuracy** of the model is defined as the proportion of the corrected classified samples over the total number of samples; this value ranges between 0 and 1, where 0 means that all samples were erroneously classified, while a value of 1 means a perfect classification. It can be defined in terms of the entries of the confusion matrix as follows:
  (5) \begin{eqnarray*}
acc = \dfrac{\sum _{k=1}^N c_{kk}}{S}
\end{eqnarray*}
- The **Matthews correlation coefficient** (MCC), proposed in [26] as a binary classification metric, was generalized to the multiclass case in 2004  [40], and it can be defined in terms of the confusion matrix as follows (see [41] for details):
  (6) \begin{eqnarray*}
MCC = \dfrac{cp \times S - \sum _{k=1}^N p_k\times t_k}{\sqrt{(S^2 - \sum _{k=1}^N p_k^2)\times (S^2-\sum {k=1}^N t_k^2) }}
\end{eqnarray*}
  where $cp=\sum _{k=1}^N c_{kk}$ is the total number of samples correctly predicted, $t_k=\sum _{i=1}^{N}c_{ik}$ is the number of times class *k* was truly occurred, and $p_k=\sum _{j=1}^N c_{kj}$ is the number of times class *k* was predicted. MCC lives in the range [−1, 1], where 1 is perfect classification, −1 is the opposite, and 0 means that the confusion matrix is all zeros but for one single column, or when all entries are equal, $c_{ij}=K \in \mathbb {N}$ [41].

#### Clustering measures

In order to assess the quality of the class separability given by the CNN, we evaluate the embeddings of the last layer (the one used to perform the classification) in the network with 3 clustering evaluation measures. These embeddings are the output from the final layer of the network for each FCGR.

- **Silhouette coefficient** [27]. Given an embedding *v* belonging to a cluster *A*, the silhouette coefficient *s*(*v*) of *v* compares the mean intracluster distance in *A* (*a*) with the mean nearest-cluster distance for *v* (*b*), that is, the closest cluster to *v* different from *A*.
  (7) \begin{eqnarray*}
s(v) = \dfrac{a-b}{\max \lbrace a,b\rbrace }
\end{eqnarray*}
  where $a = \frac{1}{|A|}\sum _{w\in A, w\ne v}^{|A|} d(v,w)$ and $b =\min _{B\ne A} \frac{1}{|B|}\sum _{w\in B}^{|B|, v \in A} d(v,w)$.
  The value of *s*(*v*) ranges between −1 (wrongly assigned) and 1 (perfect separability). For a cluster *A*, the mean silhouette coefficient of *A* is computed as the average of *s*(*v*) over all embeddings *v* ∈ *A*.
- **Calinski–Harabasz score** [28]. Given a set of embeddings *E* of size *n_E_* that has been clustered into *k* clusters, the Calinski–Harabasz score *s*, also known as the variance ratio criterion, is defined as the ratio of the between-clusters dispersion and the intercluster dispersion for all clusters (the dispersion of a group of *n* points is measured by the sum of the squared distances of the points from their centroid).
  (8) \begin{eqnarray*}
s = \dfrac{tr(B_k)}{tr(W_k)}\dfrac{n_E-k}{k-1}
\end{eqnarray*}
  where *tr*(*B_k_*) is the trace of the between-cluster dispersion matrix and *tr*(*W_k_*) is the trace (the sum of all elements in the diagonal of *W_k_*) of the within-cluster dispersion matrix, defined as follows:
  (9) \begin{eqnarray*}
W_k = \sum _{q=1}^{k}\sum _{v \in C_q} (v-c_q)(v-c_q)^T
\end{eqnarray*}
   
  (10) \begin{eqnarray*}
B_k = \sum _{q=1}^{k}n_q(c_q-c_E)(c_q-c_E)^T
\end{eqnarray*}
  where *C_q_* is the set of embeddings in the cluster *q, c_q_* is the centroid of the cluster *q*, and *c_E_* is the centroid of *E* and *n_q_* = |*C_q_*|.
  The higher the score *s* means that the clusters are dense and well separated.
- **GDV** [29]
  Given a set of *N D*-dimensional embeddings {*x*_1_, …, *x_N_*}, with *x_n_* = (*x*_*n*, 1_, …, *x_n, D_*) and a set of *L* classes {*C*_1_, …, *C_L_*}, where each *x_n_* is assigned to one of the *L* distinct classes. Consider their *z*-scored points (*s*_1_, …, *s_N_*), with *s_i_* = (*s*_*i*, 1_, …, *s_i, D_*), where $s_{n,d} = \dfrac{1}{2}\frac{x_{n,d} - \mu _d}{\sigma _d}$. Here, $\mu _d = \frac{1}{N}\sum _{n=1}^N x_{n,d}$ denotes the mean, and $\sigma _d = \sqrt{\frac{1}{N} \sum _{n=1}^{N}(x_{n,d}-\mu _d)^2}$ is the standard deviation of dimension *d*. Using the rescaled data points *s_n_* = (*s*_*n*, 1_, …, *s_n, D_*), the GDV Δ is calculated from the mean intraclass and interclass distances as follows:
  (11) \begin{eqnarray*}
\Delta = \dfrac{1}{\sqrt{D}}\Bigg [\dfrac{1}{L} \sum _{l=1}^{L} d_{intra}(C_l) - \dfrac{2}{L(L-1)}\sum _{l=1}^{L-1}\sum _{m=l+1}^{L}d_{inter}(C_l,C_m) \Bigg ]\nonumber\\ \end{eqnarray*}
  where the mean intraclass for each class *C_l_* is defined as
  (12) \begin{eqnarray*}
d_{intra}(C_l) = \dfrac{2}{N_l(N_l-1)}\sum _{i=1}^{N_l-1} \sum _{j=i+1}^{N_l}d(s_i^{(l)}, s_j^{(l)}) \end{eqnarray*}
  and the mean interclass for each pair of classes *C_l_* and *C_m_* is defined as follows:
  (13) \begin{eqnarray*}
d_{inter}(C_l,C_m) = \dfrac{1}{\sqrt{D}}\Big [\dfrac{1}{N_l N_m}\sum _{i=1}^{N_l}\sum _{j=1}^{N_m} d(s_i^{(l)}, s_j^{(l)})\Big ]
\end{eqnarray*}
  Here, *N_k_* correspond to the number of points in class *k*, and $s_i^{(k)}$ is the *i*th point of class *k*. The quantity *d*(*a, b*) is the distance between *a* and *b*; for our case, we considered the Euclidean distance. The value Δ range is between −1 (perfect separability) and 0 (wrongly assigned).

### Feature importance

After the model is trained, we can perform feature importance methods (also known as pixel attribution in case of images) to analyze the impact of each element of the FCGR in our prediction. We selected saliency maps [30] and SHAP values [31]. Saliency maps calculate the gradient of the loss function for a specific desired class with respect to the input (FCGR) elements, and the gradients are rescaled to [0, 1], where elements with values closer to 1 represent the more influential features for the input FCGR over the predicted class. SHAP values are a game-theoretic approach to explain the output of any machine learning model. It aims to explain the influence of each feature compared to the average model output over the dataset the model was trained on, and it outputs positive and negative values, where positive values push the prediction higher, and negative values push the prediction lower. Using the most relevant features from both methods over the FCGR, we aim to identify the most relevant *k*-mers for the classification of each clade.

Using these methods, we aim to analyze the most relevant *k*-mers for the classification of each clade in the trained models.

## Availability of Source Code and Requirements

- Project name: Classification of SARS-CoV-2 genome sequence with CGR and CNN
- Project home page: CouGaR-g (RRID:SCR_022952)
- Operating system(s): e.g., Platform independent
- Programming language: Python 3.10.5
- Other requirements: Python 3.10+, tensorflow 2.10.0, scikit-learn 1.1.2, tqdm 4.63.0, pandas 1.5.0, biopython 1.79, Pillow 9.0.1, matplotlib 3.5.1, shap 0.41.0, opencv-python 4.6.0.66
- License: GNU GPL

## Data Availability

The list of FASTA sequences and metadata can be downloaded from [42] after creating an account and accepting the terms of use. The data used in this study were downloaded on 4 April 2022. Trained models and results of our experiments can be downloaded from [43].

A web app version of CouGaR-g with all the trained models is available online at [44].

All supporting data and materials are available in the *GigaScience* GigaDB database [45].

## Abbreviations

CGR: chaos game representation; CNN: convolutional neural network; ENA: European Nucleotide Archive; FCGR: frequency matrix of chaos game representation; GDV: generalized discrimination value; SVM: support vector machine.

## Competing Interests

The authors declare that they have no competing interests.

## Funding

This project has received funding from the European Union’s Horizon 2020 Innovative Training Networks program under the Marie Skłodowska-Curie grant agreement No. 956229.

This project has received funding from the European Union’s Horizon 2020 Research and Innovation Staff Exchange program under the Marie Skłodowska-Curie grant agreement No. 872539.

## Authors’ Contributions

J.A.C. and S.A. wrote the code, prepared the data, and performed the experiments. J.A.C., S.A., S.C., P.B., and G.D.V. devised the methods and analyzed the results. J.A.C., S.C., and G.D.V. designed the experiments. All authors contributed to finalizing of the manuscript.

## Supplementary Material

giac119_GIGA-D-22-00106_Original_Submission

giac119_GIGA-D-22-00106_Revision_1

giac119_GIGA-D-22-00106_Revision_2

giac119_Response_to_Reviewer_Comments_Original_Submission

giac119_Response_to_Reviewer_Comments_Revision_1

giac119_Reviewer_1_Report_Original_Submission Dominik Heider -- 6/1/2022 Reviewed

giac119_Reviewer_1_Report_Revision_1 Dominik Heider -- 10/26/2022 Reviewed

giac119_Reviewer_2_Report_Original_Submission Riccardo Rizzo -- 7/19/2022 Reviewed

giac119_Reviewer_2_Report_Revision_1 Riccardo Rizzo -- 10/25/2022 Reviewed
