# Supplementary material for: Accurate and fast clade assignment via deep learning and frequency chaos game representation
Source: Gigascience. 2022 Dec 28;12:giac119. doi: 10.1093/gigascience/giac119 (PMC9795481; doi:10.1093/gigascience/giac119)
Supplement: giac119_GIGA-D-22-00106_Revision_1 [file giac119_giga-d-22-00106_revision_1.pdf]

GigaScience

Accurate and Fast Clade Assignment via Deep Learning and Frequency Chaos Game Representation

--Manuscript Draft--

**Manuscript Number:**	GIGA-D-22-00106R1
**Full Title:**	Accurate and Fast Clade Assignment via Deep Learning and Frequency Chaos Game Representation
**Article Type:**	Research
**Funding Information:**	European Union's Horizon 2020 Innovative Training Networks programme under the Marie Skłodowska-Curie (956229) — Mr Jorge Avila Cartes<br>European Union's Horizon 2020 Research and Innovation Staff Exchange programme under the Marie Skłodowska-Curie (872539) — Not applicable

Abstract:

Background:
Since the beginning of the COVID-19 pandemic there has been an explosion of sequencing of the SARS-CoV-2 virus, making it the most widely sequenced virus in the history. Several databases and tools

have been created to keep track of genome sequences and variants of the virus, most notably the GISAID platform hosts millions of complete genome sequences, and it is continuously expanding every day. A challenging task is the development of fast and accurate tools that are able to distinguish between the different SARS-CoV-2 variants and assign them to a clade.

Results:
In this paper, we leverage the Frequency Chaos Game Representation (FCGR) and Convolutional Neural Networks (CNNs) to develop an original method that learns how to classify genome sequences that we implement into CouGaR-g, a tool for the clade assignment problem on SARS-CoV-2 sequences. On a testing subset of the GISAID, CouGaR-g achieves an 96.29% overall accuracy, while a similar tool, Covidex, obtained a 77,12% overall accuracy. As far as we know, our method is the first using Deep Learning and FCGR for intra-species classification. Furthermore, by using some feature importance methods CouGaR-g allows to identify k-mers that matches SARS-CoV-2 marker variants.

Conclusions:

By combining FCGR and CNNs, we develop a method that achieves a better accuracy than Covidex (which is based on Random Forest) for clade assignment of SARS-CoV-2 genome sequences, also thanks to our training on a much larger dataset, with comparable running times.Our method implemented in CouGaR-g is able to detect k-mers that capture relevant biological information that distinguishes the clades, known as marker variants.
Availability:

The trained models can be tested online providing a FASTA file (with one or multiple sequences) at https://huggingface.co/spaces/BIASLab/sars-cov-2-classification-fcgr. CouGaR-g is also available at https://github.com/AlgoLab/CouGaR-g under the GPL.

**Corresponding Author:**	Simone Ciccolella, Ph.D.<br>Università degli Studi di Milano-Bicocca

Powered by Editorial Manager® and ProduXion Manager® from Aries Systems Corporation

	Milano, Lombardia ITALY
**Corresponding Author Secondary Information:**	
**Corresponding Author's Institution:**	Università degli Studi di Milano-Bicocca
**Corresponding Author's Secondary Institution:**	
**First Author:**	Jorge Avila Cartes
**First Author Secondary Information:**	
**Order of Authors:**	Jorge Avila Cartes
	Santosh Anand
	Simone Ciccolella, Ph.D.
	Paola Bonizzoni
	Gianluca Della Vedova
**Order of Authors Secondary Information:**	
**Response to Reviewers:**	We included a complete response to the reviewer as a PDF in the submission
**Additional Information:**	
**Question**	**Response**
Are you submitting this manuscript to a special series or article collection?	No
**Experimental design and statistics**<br><br>Full details of the experimental design and statistical methods used should be given in the Methods section, as detailed in our Minimum Standards Reporting Checklist. Information essential to interpreting the data presented should be made available in the figure legends.<br><br>Have you included all the information requested in your manuscript?	Yes
**Resources**<br><br>A description of all resources used, including antibodies, cell lines, animals and software tools, with enough information to allow them to be uniquely identified, should be included in the Methods section. Authors are strongly encouraged to cite Research Resource Identifiers (RRIDs) for antibodies, model organisms and tools, where possible.	Yes

Powered by Editorial Manager® and ProduXion Manager® from Aries Systems Corporation

Have you included the information requested as detailed in our Minimum Standards Reporting Checklist?	
**Availability of data and materials**<br><br>All datasets and code on which the conclusions of the paper rely must be either included in your submission or deposited in publicly available repositories (where available and ethically appropriate), referencing such data using a unique identifier in the references and in the "Availability of Data and Materials" section of your manuscript.<br><br>Have you have met the above requirement as detailed in our Minimum Standards Reporting Checklist?	Yes

Powered by Editorial Manager® and ProduXion Manager® from Aries Systems Corporation

Click here to access/download;LaTeX - Main Document (TeX file);main_gs.tex

OXFORD (GIGA)^n SCIENCE

GigaScience, 2017, 1–11

doi: xx.xxxx/xxxx
Manuscript in Preparation
Paper

PAPER

Accurate and Fast Clade Assignment via Deep Learning and Frequency Chaos Game Representation

Jorge Avila Cartes[1], Santosh Anand[1], Simone Ciccolella[1], Paola Bonizzoni[1] and Gianluca Della Vedova[1,*]

[1]Department of Computer Science, Systems and Communications. University of Milano–Bicocca, Italy

*gianluca.dellavedova@unimib.it

Abstract

Background: Since the beginning of the COVID-19 pandemic there has been an explosion of sequencing of the SARS-CoV-2 virus, making it the most widely sequenced virus in the history. Several databases and tools have been created to keep track of genome sequences and variants of the virus, most notably the GISAID platform hosts millions of complete genome sequences, and it is continuously expanding every day. A challenging task is the development of fast and accurate tools that are able to distinguish between the different SARS-CoV-2 variants and assign them to a clade.
Results: In this paper, we leverage the Frequency Chaos Game Representation (FCGR) and Convolutional Neural Networks (CNNs) to develop an original method that learns how to classify genome sequences that we implement into CouGaR-g, a tool for the clade assignment problem on SARS-CoV-2 sequences. On a testing subset of the GISAID, CouGaR-g achieves an 96.29% overall accuracy, while a similar tool, Covidex, obtained a 77,12% overall accuracy. As far as we know, our method is the first using Deep Learning and FCGR for intra-species classification. Furthermore, by using some feature importance methods CouGaR-g allows to identify k-mers that matches SARS-CoV-2 marker variants.
Conclusions: By combining FCGR and CNNs, we develop a method that achieves a better accuracy than Covidex (which is based on Random Forest) for clade assignment of SARS-CoV-2 genome sequences, also thanks to our training on a much larger dataset, with comparable running times. Our method implemented in CouGaR-g is able to detect k-mers that capture relevant biological information that distinguishes the clades, known as marker variants.
Availability: The trained models can be tested online providing a FASTA file (with one or multiple sequences) at `https://huggingface.co/spaces/BIASLab/sars-cov-2-classification-fcgr`. CouGaR-g is also available at `https://github.com/AlgoLab/CouGaR-g` under the GPL.

Key words: Chaos Game Representation, Convolutional Neural Networks, Classification of genome sequences, SARS-CoV-2, GISAID clades, k-mer frequency, deep learning.

Introduction

The global coordination in combating the COVID-19 pandemic has led to the sequencing of one of the largest amount of viral genomic data ever produced. All this data is stored in publicly available archives, such as the European Nucleotide Archive (ENA) and GISAID [1], currently having more than 9.6 million sequenced genomes, classified in *variants*, *clades*, and *lineages*.

The SARS-CoV-2 virus has evolved since its discovery, and the currently available phylogenies describing its evolutionary history [2] show more than 2000 different genomes, divided into lineages. Since the phylogeny is fairly stable and the main (existing) *lineages*, i.e. the lines of descent, have been identified, a natural and interesting problem is to quickly find, given a sequence, the *clade* to which it belongs, i.e. a group of descendants sharing a common ancestor [3]. Fast and efficient solutions to the clade assignment problem would help in tracking current and evolving strains and it is crucial for the surveillance of the pathogen. This classification problem has been

Compiled on: October 17, 2022.
Draft manuscript prepared by the author.

attacked with machine learning approaches [4, 5, 6] using the Spike protein amino acid sequence to drive the classification step.

In this paper we propose a method for classifying SARS-CoV-2 genome sequences based on Chaos Game Representation (CGR) [7]: a deterministic bi-dimensional representation of a DNA sequence, also called CGR encoding, that can be easily obtained from the genome sequences. The CGR encoding of a sequence has two fundamental properties: it is deterministic, that is there is a unique CGR encoding of each sequence, and reversible, hence the original sequence can be recovered from its representation [8].

A strongly related approach, known as Frequency matrix of Chaos Game Representation (FCGR) [9, 8], starts from the k-mers (the substring of length k) of the string we want to represent resulting in the the notion of k-th order FCGR [10]. The *k-th order FGCR* of a sequence s is a $2^k \times 2^k$ matrix whose elements are the number of occurrences, i.e. the frequencies, of each k-mer in s, where each frequency is stored in the specific and distinct position for each k-mer. Note that the matrix shape depends on the fact that the sequence s is on a 4-symbol alphabet. In essence, the FCGR is an alternative ordering of the histogram for all the k-mers (for a fixed integer k). Deep Learning and FCGR have been used to evaluate the drug resistance for protein sequences of HIV [11]; for multi-class classification task to identify the source organism for a given protein [12] — in this case the FCGR has been extended to encode sequences in the protein alphabet — and to predict antimicrobial resistance of different drugs in *E. Coli* [13]. The FCGR has also been used for unsupervised clustering of DNA sequences of several species [14] by using dense neural networks, where the input of these networks must be a 1-dimensional vector. In this case, the 2-dimensional FCGR representation of the sequences must be flattened and cannot be fully exploited. For an extensive review on CGR and its applications in bioinformatics, we refer the reader to [15].

Subtyping Sars-Cov-2 sequences has been addressed in the literature with bioinformatics pipelines that require the alignment to a reference genome [2] [16], and also with machine learning approaches aiming to skip the alignment step [17]. Furthermore, an early approach to construct phylogenetics trees within SARS-CoV-2 strains and closely related species was proposed in [18] using FCGR as embedding for a Hierarchical Agglomerative Clustering. In a similar fashion, FCGR was explored along with other techniques as embedding for the identification of homologies between different known and emerging viruses in [19]. Convolutional Neural Networks (CNNs) [20, 21], showed outstanding results in the well-known Imagenet classification problem [22]. To the best of our knowledge, only two works have used CNNs and FCGR for the classification of DNA sequences. In [23] a simplification of the network reported in [21] was used to classify different taxonomic categories with a dataset of 3,000 sequences (1200–1400 long). A comparison with Support Vector Machines (SVM), showed that CNNs improve over SVM when using a fragment (500bp) of the sequences. In [24], a CNN was proposed for the classification of a dataset of ≈ 660 sequences from eleven phylogenetic families reporting a test accuracy of 87%.

In this paper we leverage the FCGR representation of genomic sequences and CNN power to perform intra-species classification of viral DNA genome sequences, using SARS-CoV2 as our case of study and GISAID clades as our labels. Observe that in this problem the CNN classifies a dataset that is at least two order of magnitude larger than the one considered in the above mentioned papers. Another work that has tackled the clade assignment problem is Covidex [25], a web app tool based on Random Forest and k-mer frequencies: to the best of our knowledge this is the most recent work facing our problem. Notice that almost the entire phylogenetics literature deals with inter-species classification, where the distance between possible cluster centroids is larger, hence the classification problem is easier. We propose to use a residual neural network [26] (ResNet50) for the classification of DNA sequences into 11 GISAID clades, using a dataset of two orders of magnitude larger (153K sequences for training) than those analyzed in the above cited works (about 3000 sequences in [23]).

Classification metrics (accuracy, Matthews Correlation Coefficient [27], precision, recall and f1-score) and analysis of the separability of the embeddings generated by the classification layer (Silhouette Coefficient [28], Calinski-Harabasz Score [29], and Generalized Discrimination Value (GDV) [30]) are analyzed for each model. Using the fact that each feature in the FCGR is uniquely related to a k-mer, we aim to analyze if the most relevant k-mers identified by feature importance methods (Saliency Maps [31] and Shap Values [32]) are related to mutations defining each clade.

We trained four models, one for each value of $k \in \{6, 7, 8\}$. All models performed very similarly, with $k = 8$ being the best one, achieving an overall accuracy of 96.22% in the test set, and the best classification metrics (0.948 for Silhouette Coefficient, 174, 736.1 for Calinski-Harabasz and −0.718 for GDV). Three clades (O, GR and GRY) reported the lowest f1-score for all the trained models. Since GR is a close ancestor of GRY and these two clades share many mutations, they are confused with each other. For clade O, mispredictions are among most of the clades.

Using the 20 most relevant k-mers identified by Saliency Maps, we were able to achieve a similar performance than our CNNs models using SVM for $k \in \{6, 7, 8\}$. Finally, to access the performance of our models w.r.t. other approaches, we compare our results with Covidex [25] the only recent tool that we found in the literature solving the clade assignment problem. Our results show that our models outperform Covidex in all clades and reported metrics (accuracy, precision, recall and f1-score).

Background

The Chaos Game Representation for encoding DNA/RNA sequences is formally defined as:

Definition 1 (Chaos Game Representation (CGR)) *Let $s = s_1 \dots s_n \in \{A, C, G, T\}^*$ be a sequence. Then the CGR encoding of the sequence s is the bi-dimensional representation of the ordered pair (x_n, y_n) which is defined iteratively as*

$$(x_i, y_i) = \frac{1}{2}\Big((x_{i-1}, y_{i-1}) + g(s_i)\Big), \text{ if } i \geq 1 \tag{1}$$

where $(x_0, y_0) = (0, 0)$ and,

$$g(s_i) = \begin{cases} (1,1) & s_i = A \\ (-1,1) & s_i = C \\ (-1,-1) & s_i = G \\ (1,-1) & s_i = T \end{cases} \tag{2}$$

Note that each point (x_i, y_i) obtained with the above encoding represents the i-long prefix of the sequence s. Also, all the CGR encodings are points inside the square with vertices given by the values of the function g. In particular, the encoding of all prefixes that shares the last character will be placed in the same quadrant, all prefixes that shares the two last characters, will be placed in the same sub-quadrant, and so on. This property results in a fractal structure of the representation.

Missing bases can be problematic to encode, since the $g(\cdot)$ function is not defined in that case, we used the notion of frequence matrix CGR [9, 8], which has the added benefit of allowing us to manage k-mers instead of strings of arbitrary length.

Definition 2 (Frequency matrix of Chaos Game Representation) *Let $s = s_1 \ldots s_n \in \{A, C, G, T, N\}^*$ be a sequence, and let k be an integer. Then the frequency matrix of CGR, in short FCGR, of the sequence s is a $2^k \times 2^k$ bi-dimensional matrix $F = (a_{i,j}), 1 \leq i, j \leq 2^k, i, j \in \mathbb{N}$. For each k-mer $b \in \{A, C, G, T\}^k$, we have an element $a_{i,j}$ in the matrix F, that is equal to the number of occurrences of b as a substring of s. Moreover, the position (i, j) of such element is computed as follows:*

$$
\begin{aligned}
i &= \quad 2^k - \lceil 2^{k-1}(x+1) \rceil + 1 \\
j &= \quad \lceil 2^{k-1}(y+1) \rceil
\end{aligned}
$$

where (x, y) is the CGR encoding for the k-mer b.

Note that the FCGR is defined for a DNA sequence with unknown nucleotide, denoted by N — where k-mers with an N are simply excluded in the counting process— while the *CGR* encoding is well-defined only when all nucleotides are known. To explicitly mention the dimension of the FCGR, we will refer to this as the k-th order FCGR.

Classification of viral sequences of DNA

We are given a phylogeny over the possible viral strains, partitioned into classes: each class c of such partition $\mathcal{C}$ is a *clade* of the tree. More precisely, a clade is a group of related organisms descended from a common ancestor [3], in other words a clade is a subtree of a phylogeny that consist of an ancestral lineage and all its descendants.

Given a genome sequence, that is a string $s \in \{A, C, G, T, N\}^*$, we determine the original clade in $\mathcal{C}$ from which the genome sequence is originated; however the genome sequence s might not have been previously observed. In any case the sequence will be assigned to a putative clade. To solve this problem, we propose a supervised learning model based on Convolutional Neural Networks (CNN) [21], using FCGR as inputs.

Data Description

The dataset for this experiment was downloaded from GISAID. By the time of our access to GISAID [1] there were around 10 million sequences.

In order to undersample the available data, we first dropped all the rows in the metadata without information in the columns `Virus name`, `Collection Date`, `Submission Date`, `clade`, `Host` and `Is complete?`, then we built a `fasta_id` identifier from the metadata as a concatenation of the columns `Virus name`, `Collection Date` and `Submission Date`.

For each clade, we randomly selected $20,000$ sequences considering only those rows where the `Host` column has value "Human" — clades L, V, and S have less than $20,000$ sequences available, in these cases all sequences have been selected.

As a result of the above procedure, we obtained $191,456$ sequences among the 11 GISAID clades (S, L, G, V, GR, GH, GV, GK, GRY, O, and GRA) over the 12 available, we excluded the clade GKA from our study since there were only 81 sequences reported in the metadata. The undersampled dataset was randomly split into train, validation and test sets in $80 : 10 : 10$ proportion, preserving the same proportion of clades (labels) in each set. The distribution of the clades over the datasets is given in Table 1

Analyses

In this section we present the experimental setup, the dataset used to train and test each model, and clustering and classification metrics. We train one model for each $k \in \{6, 7, 8\}$ and we complement the study of the accuracy of each model (compared against Covidex [25]) with an analysis of the most relevant k-mers for the classification of each clade using Saliency Maps and Shap.

For this experiment we choose $k \in \{6, 7, 8\}$ and sequences from 11 GISAID clades: S, L, G, V, GR, GH, GV, GK, GRY, O and GRA.

Experimental setup

All experiments are conducted using a Intel(R) Core(TM) i5-10400 CPU @ 2.90GHz, x86_64, 32 GB RAM and a graphic card NVIDIA GeForce RTX 3060. The implementation is done in Python 3.10.5. Tensorflow 2.10.0 [33] was used for training the CNN and scikit-learn 1.1.12 [34] to compute classification metrics and clustering evaluation (except for Generalized Discrimination Value that was implemented). All code is available online for reproducibility [2].

Clade	Train	Val	Test	Total	Available
S	14,298	1,788	1,788	17,874	17,874
L	5,154	644	644	6,442	6,442
G	15,999	2,000	2,000	20,000	408,552
V	5,713	714	714	7,141	7,141
GR	16,000	2,000	2,000	20,000	625,662
GH	16,000	2,000	2,000	20,000	547,792
GV	16,000	2,000	2,000	20,000	182,248
GK	16,000	2,000	2,000	20,000	4,170,758
GRY	16,000	2,000	2,000	20,000	944,876
O	16,000	2,000	2,000	20,000	55,400
GRA	16,000	2,000	2,000	20,000	2,833,863
	153,164	19,146	19,146	191,456	9,800,608

Table 1. Distribution of the number of sequences selected for train, validation and test sets by each clade. The final dataset for the 11 clades was split in a $80 : 10 : 10$ proportion for train, validation, and test sets.

Model training

Each model was set to be trained for 50 epochs with a batch size of 32 using Adam optimizer [35] with learning rate 0.001 (the default parameters in keras). The validation loss was monitored after each epoch to save the best trained weights, reducing the learning rate with a patience of 8 epochs and a factor of 0.1, and by an early stopping in case the metrics do not improve after 12 epochs.

We show the accuracy (average of the 5 Repeated fold cross validation) of the train and validation sets for $k = 8$ in Figure 1. For $k = 6$ and $k = 7$ the training is more unstable for the first epochs, but it behaves similar to $k = 8$ in the later epochs, i.e. training and validation metrics are similar.

1 April 04, 2022. https://www.gisaid.org/

2 https://github.com/AlgoLab/CouGaR-g

k-mer	Dimensions	Features	Size (GiB)	Time per epoch (min)
6	(64,64)	4,096	6.6	4:05
7	(128,128)	16,384	24.1	8:21
8	(256,256)	65,536	94.2	24:50

Table 2. For each k, the dimension of the FCGR, its number of features (4^k), the amount of memory required to store the selected dataset of 191,456 sequences as FCGR, and the average training time per epoch are reported in the table. The number of features and the space increase exponentially w.r.t k.

k-mer	Accuracy	MCC
6	0.953714 ± 0.001589	0.948792 ± 0.001813
7	0.959856 ± 0.001740	0.955566 ± 0.001910
8	**0.962175 ± 0.002829**	**0.958211 ± 0.003141**

Table 3. Accuracy and Matthews Correlation Coefficient in the set for each of our models. Each metric ($\mu \pm \sigma$) is reported by its average (μ) and standard deviation (σ) from a 5 RepeatedFold cross validation process. For both, accuracy and MCC the model increases with the value of k. Going from k=6 to k=8 increases accuracy in 0.84%, and MCC in 0.94%. In **bold** the highest value of each metric.

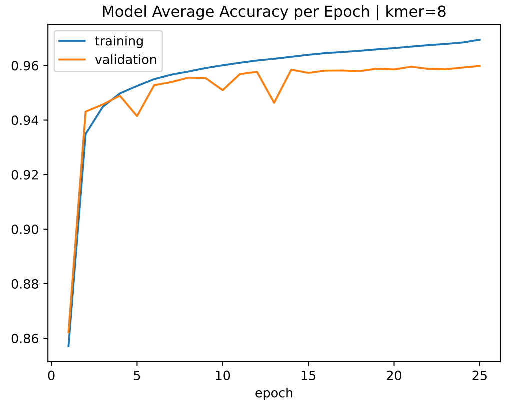

Figure 1. Average accuracy in the training and validation sets for our model with $k = 8$. The best model (final weights) is set as the one with the lowest validation loss, achieved at epochs 24 ± 5 for $k = 8$ (from a 5 RepeatedFold cross validation process). All models were trained for 50 epochs using an early stopping of 12 epochs based on the validation loss (hence, not all of them ran for 50 epochs).

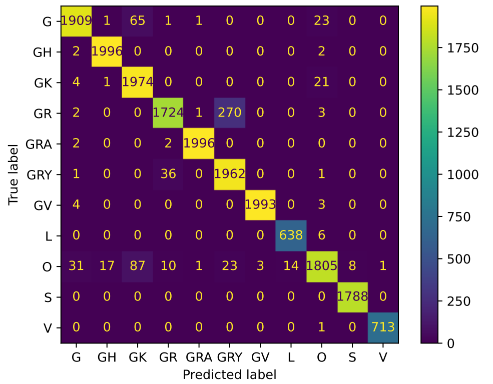

Figure 2. Confusion matrix for the test set for one of the trained models with $k = 8$ (from a 5 RepeatedFold cross validation process). All the models are able to correctly classify more than 98% of the sequences for all clades except for G, GR, GRY, and O. Most of the incorrectly classified sequences of GR and GRY are confused between them, which makes sense since they are evolutionary related. For the G clade, the incorrectly classified sequences are shared between clades GK and O. For the clade O, the incorrectly classified sequences are predominantly assigned to clades G, GH, GK, and GRY.

The architecture used in this experiment is the same for all k (ResNet50 [26]), we only changed the input size. Originally, this architecture was designed for inputs of size ($224 \times 224 \times 3$), which led us to the assumption that this architecture could be more suitable for $k = 8$. Notice that our sequences are $\approx 29,000bp$ long, which means that our input FCGR for $k = 8$ is very sparse, since from an n-long sequence we can count $n-k+1$ k-mers, it means that (in the case where all k-mers are different) we have at most $29,000$ k-mers, at least 55% of the elements of the FCGR are 0 for $k = 8$. In Table 2 a comparison of the number of features for each k and the training time per epoch in our experiments is detailed.

Classification results

After each model is trained the precision and recall for the test set are computed for each clade using the best trained weights (lowest loss in the validation set), achieved at epochs 24 ± 5, 27 ± 8, and 20 ± 3 for $k = 6$, $k = 7$, and $k = 8$, respectively. In our case, we assign each sequence to the clade with highest score. Precision, recall and f1-score are shown in Table 4.

Precision and recall are very similar among all the trained models, with small improvements when k increases, 5 out of 11 clades have f1-score greater than 99% in our best model ($k = 8$). Most notable differences in the performance can be seen in clades GR and GRY, which present the lowest (and under 90%)reported recall and precision in each model, respectively. Moreover, from the confusion matrices (see Fig. 2) we can see that misclassified sequences that belong to clades GR and GRY, are confused between them, this can be explained since clade GRY is originated from clade GR. For the other clades, most of the misclassified sequences are predicted as (or belong to) clade G, that is the former one. Clade O exhibits the second lowest recall, where the misclassified sequences are assigned predominantly to clades G, GH, GK, and GRY.

Comparison with the literature

We compare our results against Covidex [25], a tool that classify Sars-CoV-2 sequences into three nomenclatures: GISAID, Nexstrain and Pango lineages. Using a different model for each task, all based on Random Forest and 6-mers as input. The reported accuracy are 97,77%, 99,52% and 96,56% for GISAID, Nextstrain and Pango models, respectively. They also trained the models using 7-mers, but they claim that it only produced slightly better results in terms of accuracy but with more than doubling the computation time [25].

The input for Covidex is a vector with the normalized counting of the frequencies for all 4^k k-mers. Our input, the FCGR also considers all k-mers but in a bi-dimensional matrix. The main difference between both approaches is the model behind it, while Covidex uses Random Forest to perform the classification, we take advantage of the CNNs and use a 2-dimensional input, the FCGR. Notice that using the FCGR with any other classical Machine Learning method implies to convert the FCGR into a vector, and hence, the loss of the 2-dimensional structure.

Since our model is trained using GISAID clades, we only compare to those results. In Covidex, they used 10 clades: S, L, G, V, GR, GH, GV, GK, GRY and O. In our case, we included GRA since there were enough available sequences by the time of our experiments, but this is not considered in the comparison.

k-mer	6			7			8		
Clade	Precision	Recall	F1score	Precision	Recall	F1score	Precision	Recall	F1score
S	99.4 ± 0.2	99.6 ± 0.2	99.5 ± 0.1	99.7 ± 0.1	99.6 ± 0.1	99.7 ± 0.1	99.8 ± 0.2	99.7 ± 0.3	**99.8 ± 0.1**
L	98.3 ± 0.4	97.7 ± 0.6	98.0 ± 0.3	98.7 ± 0.4	99.0 ± 0.2	**98.9 ± 0.2**	98.0 ± 0.4	99.5 ± 0.3	98.7 ± 0.2
G	95.8 ± 0.7	94.7 ± 0.8	95.2 ± 0.4	97.1 ± 0.4	95.1 ± 0.4	96.1 ± 0.3	97.3 ± 0.8	95.9 ± 0.7	**96.6 ± 0.1**
V	99.1 ± 0.4	99.2 ± 0.4	99.1 ± 0.2	99.5 ± 0.5	99.4 ± 0.3	99.4 ± 0.3	99.6 ± 0.5	99.6 ± 0.2	**99.6 ± 0.3**
GR	91.7 ± 2.0	85.9 ± 1.7	88.7 ± 0.4	92.4 ± 1.3	87.5 ± 1.4	89.8 ± 0.2	93.9 ± 2.3	86.6 ± 1.7	**90.1 ± 0.7**
GH	98.6 ± 0.3	99.5 ± 0.1	99.0 ± 0.2	98.9 ± 0.2	99.7 ± 0.1	**99.3 ± 0.1**	98.8 ± 0.2	99.8 ± 0.1	**99.3 ± 0.1**
GV	99.5 ± 0.3	99.6 ± 0.2	99.5 ± 0.1	99.7 ± 0.1	99.6 ± 0.1	99.7 ± 0.1	99.6 ± 0.1	99.8 ± 0.1	**99.7 ± 0.0**
GK	91.8 ± 0.4	97.6 ± 0.5	94.6 ± 0.2	92.2 ± 0.5	97.7 ± 0.2	94.9 ± 0.4	92.7 ± 0.2	98.1 ± 0.8	**95.3 ± 0.4**
GRY	86.4 ± 1.3	93.2 ± 2.4	89.7 ± 0.6	87.8 ± 1.1	93.9 ± 1.4	90.7 ± 0.3	87.1 ± 1.5	95.5 ± 2.0	**91.0 ± 0.7**
O	94.3 ± 0.8	86.8 ± 0.2	90.4 ± 0.4	95.0 ± 0.6	89.2 ± 0.8	92.1 ± 0.6	96.7 ± 0.4	88.8 ± 1.2	**92.6 ± 0.7**
GRA	99.7 ± 0.1	99.8 ± 0.1	99.8 ± 0.1	99.9 ± 0.1	99.7 ± 0.1	**99.8 ± 0.0**	99.8 ± 0.1	99.8 ± 0.1	99.8 ± 0.1

Table 4. Precision, recall, and f1-score. Each of our models is represented by length of the the k-mers used to generate the FCGR. Two clades, GR and GRY present deviations greater than 1% in their precision and recall for all values of k. In **bold** the highest F1score for each clade and k. Each metric ($\mu \pm \sigma$) is reported by its average (μ) and standard deviation (σ) from a 5 RepeatedFold cross validation process.

	Report			Test		
Clade	Prec.	Rec.	F1score	Prec.	Rec.	F1score
S	0.998	1	0.999	**0.988**	**0.995**	**0.991**
L	0.997	1	0.999	0.859	**0.979**	0.915
G	0.993	0.984	0.989	0.811	0.852	0.831
V	1	1	1	0.958	**0.985**	**0.971**
GR	0.945	0.915	0.930	0.379	0.760	0.506
GH	0.995	0.999	0.997	0.957	**0.987**	**0.972**
GV	0.996	0.999	0.997	**0.980**	**0.995**	**0.988**
GK	0.977	0.995	0.986	0.925	0.833	0.877
GRY	0.920	0.961	0.940	0.732	0.763	0.747
O	0.994	0.959	0.976	0.722	0.658	0.688

Table 5. Precision, recall, and f1-score for Covidex. The Report part is taken from the Supplementary material of [25]. The Test part has the precision, recall, and f1-score obtained by Covidex on our test set, restricted to the 10 clades (17,146 sequences) analyzed in [25]. We found significant differences between Covidex and our trained models in the Test metrics (see Table 4). In particular, the most notorious differences w.r.t f1-score, ranging from 8.4%–42.4% are found for clades L (−8.4%), G (−15.8%), GR (−42.4%), GK (−10.9%), GRY (−19.3%) and O (−28.8%), while for clades S (−0.8%), V (−2.9%), GH (−2.5%) and GV (−0.9%), we can observe a decrement on the reported f1-score ranging from 0.8%–2.9%. Metrics in **bold** in the Test part are those which **did not decrease** more than 3% w.r.t the reported metrics.

For Covidex, the model for the GISAID nomenclature was trained with 66,126 sequences and tested on 13,230. Since Covidex is made available as an user app for any SARS-Cov2 sequence, we used the app over our test dataset to compare the results. We tested Covidex on our test dataset of 17,146 sequences (excluding the 2000 sequences from GRA clade). Achieving a 77,12% of accuracy, more than a 18% lower than all our trained models and 20,65% lower than their reported accuracy. The reported precision, recall and f1-score, as well as the test results over our selected dataset can be seen in Table 5. We found that the reported f1-score of Covidex is quite distant for the one we obtained in our test dataset for clades L (−8.4%), G (−15.8%), GR (−42.4%), GK (−10.9%), GRY (−19.3%) and O (−28.8%), while for clades S (−0.8%), V (−2.9%), GH (−2.5%) and GV (−0.9%), we can observe a decrement on the reported f1-score ranging from 0.8% to 2.9%. Our models (see Table 4) exhibit better performance than Covidex in all clades and metrics on our test set, with similar results only on clades S and GV. We did not perform an extensive comparison of the running times since both tools classify a genome sequence in less than a second (on $k = 8$, our tool took 0.15 seconds in average).

k-mer	Silhouette	Calinski-Harabasz	GDV
6	0.939 ± 0.007	145,879.214 ± 18,132.428	-0.712 ± 0.003
7	0.948 ± 0.003	163,926.767 ± 8,638.391	-0.717 ± 0.002
8	0.948 ± 0.006	174,736.086 ± 22,554.904	-0.718 ± 0.003

Table 6. Clustering metrics for our trained models. Each metric is computed using the output of each model and the predicted clade (that is, the clade that achieves the highest score by our model) in the test set. Each model is represented by the length of the k-mers used to generate the FCGR. For the Silhouette score, the closest to 1 the better. For the Calinski-Harabasz score, larger values are better. For the GDV score, the closest to −1, the better. All models exhibit comparable separability of the clusters. Each metric ($\mu \pm \sigma$) is reported by the average (μ) and standard deviation (σ) in the from a 5 RepeatedFold cross validation process.

Clustering results

We evaluate the embeddings of the last layer of each trained model using the Silhouette Coefficient, Calinski-Harabasz score and Generalized Discrimination Value (GDV). These results are shown in Table 6. We can observe that the model for $k = 9$ is the best one among all metrics, however, all trained models exhibit a very similar separability based on Silhouette and GDV.

Relevant k-mers for the classification of each clade.

The purpose of this experiment is to study if a set of the most relevant k-mers (based on feature importance methods) are informative enough to a SVM to perform similarly than the trained CNNs (that uses FCGR as input, and hence all the 4^k possible k-mers).

Using Saliency Maps and Shap Values, we can evaluate the contribution of each element of a FCGR in the classification, for each model. From each one of these feature attribution methods we can obtain an ordered list of all k-mers. For each clade, we use the centroid FCGR of all correctly classified sequences in the test set, then we use each centroid FCGR to identify the most relevant k-mers for each clade and then train a SVM using the N most relevant k-mers (for different values of $N \in \{1, 2, 3, 4, 5, 10, 15, 20, 25, 30, 35, 40, 45, 50\}$) and their respective frequencies as input.

The same training and test sets used for the CNNs were used for the SVM. The results of the accuracy in the test set for the different values of N are shown in Figures 6 and 5. We can observe that k-mers identified by Saliency Maps are more informative than those identified by Shap Values, since for $N = 20$,

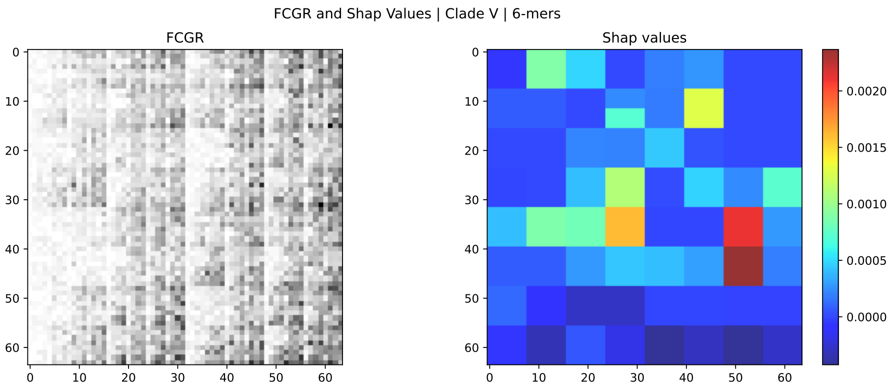

Figure 3. FCGR image (left) and Shap Values (right) of the centroid FCGR for the clade V ($k = 6$). The FCGR image is obtained rescaling the frequencies in the FCGR to a gray-scale range of 8 bits ([0,255]), an inversion of colors is performed to visualize higher values as black squares and lower values as white. Shap Values represent the importance of the features in the FCGR, the higher the value (red) the more important is the feature. Each feature (pixel) in the FCGR corresponds to a k-mer.

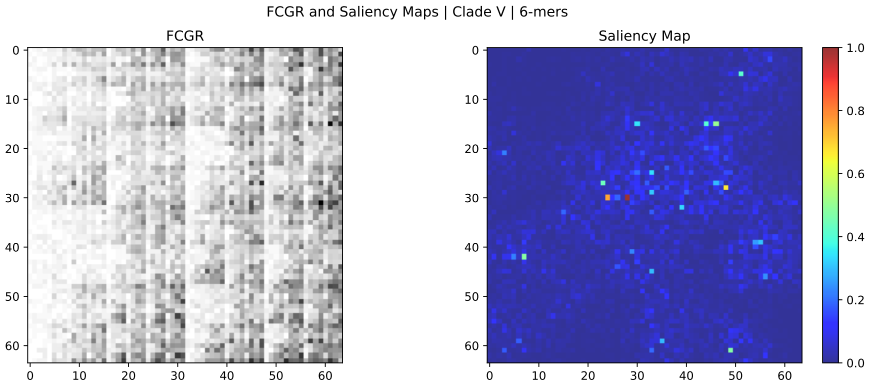

Figure 4. FCGR image (left) and Saliency Map (right) of the centroid FCGR for the clade V ($k = 6$). The FCGR image is obtained rescaling the frequencies in the FCGR to a gray-scale range of 8 bits ([0,255]), an inversion of colors is performed to visualize higher values as black squares and lower values as white. Saliency Map represent the importance of the features in the FCGR, the higher the value (red) the more important is the feature. Each feature (pixel) in the FCGR and Saliency Map corresponds to a k-mer.

we obtain similar accuracy in the test set for $k = 6, 7, 8$ compared to CNN (96–97%), while in the case of Shap Values, this accuracy is only achieved by $k = 7$ with $N = 35$. Notice that using $N = 20$, we are considering a small number of all possible k-mers (0.49% for $k = 6$, 0.12% for $k = 7$ and 0.03% for $k = 8$).

Matching relevant k-mers to mutations.

Using the reference genome employed by GISAID (EPI_ISL_402124) [3] and the list of marker variants [4] for each GISAID clade with respect to this reference, we evaluated how many k-mers among the 50 chosen ones by Saliency Maps and Shap Values actually matched any of the reported marker variants. A summary is shown in Table 7.

The results shown that the most relevant k-mers selected using Saliency Maps match several of the reported marker variants (46 matches for $k = 6$, 51 for $k = 7$, and 11 for $k = 8$). On the other hand, the ones chosen by Shap Values barely match with the mutation (3 for $k = 6$), suggesting that Saliency Maps could provide a richer explainability of the model from a biological perspective.

3 https://www.gisaid.org/resources/hcov-19-reference-sequence/
4 https://www.gisaid.org/resources/statements-clarifications/clade-and-lineage-nomenclature-aids-in-genomic-epidemiology-of-active-hcov-19-viruses/

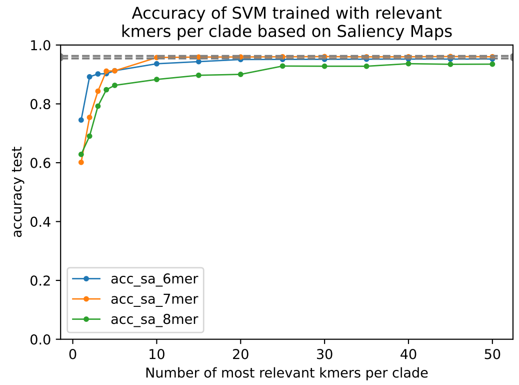

Figure 5. Accuracy of test set for SVM trained models using only the most N relevant k-mers for each clade ($N \in \{1, 2, 3, 4, 5, 10, 15, 20, 25, 30, 35, 40, 45, 50\}$). The relevant k-mers are selected using Saliency Maps on the centroid of the correctly classified FCGR for each clade and model. The same train and test datasets used for the trained CNNs are used for the SVM. The SVM trained with 20 most relevant k-mers identified by Saliency Map, for $k \in \{6, 7\}$ achieves an accuracy in the test set ($\approx$ 96%) that is in the range of the minimum and maximum accuracies (see Table 3) obtained by our trained CNNs (the gray dashed band represents the minimum and maximum accuracy for the trained CNNs).

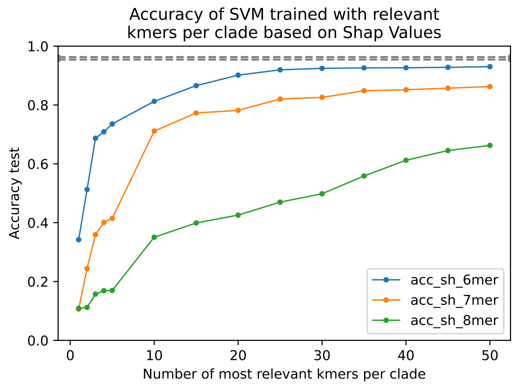

Figure 6. Accuracy of test set for SVM trained model using only the most N relevant k-mers for each clade ($N \in \{1, 2, 3, 4, 5, 10, 15, 20, 25, 30, 35, 40, 45, 50\}$). The relevant k-mers are selected using Shap Values on the centroid of the correctly classified FCGR for each clade and model. The same train and test datasets used for the trained CNNs are used for the SVM. The SVM trained with the 30 most relevant (or more) 6-mers identified by Shap Values, achieves the closest accuracy (92, 44%) to the ones obtained by our trained models (see Table 3). When k increases, the accuracy always decreases (for the same number of relevant k-mers), which can be explained since when k increases the total number of possible k-mers increases exponentially.

k-mer	Saliency Maps	Shap Values
6	46	3
7	51	0
8	11	0

Table 7. Summary of matches between the 50 most relevant k-mers (from Saliency Maps and Shap Values) and the list of marker variants reported by GISAID for each clade. The k-mers obtained by Saliency Maps are able to match several mutations and the matches decrease when k increases, but the ones from Shap Values only reported 3 matches, for $k = 6$.

Discussion

In this work we have shown that FCGR can be used to classify DNA sequences. Most notably, we have used FCGR to assign SARS-CoV-2 genome sequences to its GISAID strain by running a CNN on 191, 456 genome sequences (80% training set, 10% validation set, and 10% test set). In particular, the 8-th order FCGR achieved a test accuracy of 96.22%. The majority of misclassified sequences are shared between two strongly related strains, GR and GRY (GR is a close ancestor of GRY).

We decided to exclude transfer learning from our experiments after trying this approach without success on 8-mers. For this trial we used pre-trained weights from the Imagenet dataset using ResNet50 architecture, where the backbone weights were frozen, and three dense layers were included at the top of it for the classification.

We have assessed the influence of the length k of the substrings (k-mers) used to build the FCGR, showing that values between 6 and 8 lead to very similar results, with less than 1% of difference in both accuracy and MCC on the same test set. However, when increasing the value of k, the training time for the model and the memory required to save the FCGRs increases exponentially. For $k = 6$ each epoch required 4:05 minutes and 6.6GB of memory, while for $k = 8$ it required 24:50 hour and 94.2GB. However, FCGRs show a fractal structures; this suggests that we might couple increasing k with using only a portion of the FCGR.

We compare our results with Covidex, a Random Forest based tool that classify sequences on GISAID clades based on k-mers frequencies. Under the same test set, our results show that our models outperform Covidex in all clades and reported metrics (accuracy, precision, recall and f1-score). Moreover, we found that the reported precision, recall and f1-score of Covidex are quite different for all clades but S and GV in our test set, exhibiting a decreasing in the f1-score metric up to 42.4%.

We have used Saliency Maps and Shap to identify relevant k-mers, looking for matches with the marker variants reported for each strain. Using the k-mers obtained by Saliency Map, we found 46, 51, and 11 matches for $k = 6, 7, 8$, respectively. While, for the k-mers identified by Shap, only 3 matches were found for $k = 6$. A possible direction for future works is to explore other existing methods (*e.g.* Lime [36], GradCAM [37], DeepLIFT [38]) that might be suitable in explaining the decisions of the model.

Classifying genome sequences introducing the assembly bias includes more factors to take care of, since any classification depends on the specific assembly pipeline that has been used. To lessen this possible problem, we should study a related problem, where we classify read samples instead of fully assembled genomes. This new problem is more complex, since different regions of the viral genome can have different coverage — hence impacting the frequencies — and reads needs to be cleaned from both errors and contamination artifacts (the latter might be attacked with specialized tools like KMC3 [39]).

We did not perform an extensive comparison of the running times since both tools classify a genome sequence in less than a second.

Potential implications

This paper shows how to couple Frequency Chaos Game Representation with a deep neural network that is especially suited to represent images, such as a CNN, to predict clade assignment. Since FCGR is a simple and intuitive representation of a set of k-mers, we expect this combination to find applications in several other problems that are currently attacked with approaches based on k-mers.

Methods

We use the k-th order FCGR representation for each sequence. In order to obtain this representation, we need to count the k-mers in each sequence and to put those frequencies in the FCGR based on the CGR encoding.

Before feeding the FCGR to the model, we rescale its elements to values between 0 and 1 for stability of the learning process. To do so, we divide each FCGR element-wise by the maximum value in the FCGR. It is worth mentioning that other preprocessing steps were taken into consideration but were ultimately excluded because found empirically worse.

Due to the huge amount of data available for most of the clades, we undersampled at most 20.000 sequences per clade to perform our experiments, nevertheless, for some clades only a portion of it was available (see table representativity). In order to overcome the unbalance in the undersampled dataset, we decided to use a weighted binary cross-entropy loss function (instead of oversampling the underrepresented classes), where the cost associated with a class c is inversely proportional to its representativity in the training set.

Model architecture

We choose a residual neural network, ResNet50 [40] as our CNN, adapted for k-th order FCGR, i.e. with input size equal to $(2^k \times 2^k \times 1)$, and output size equal to the number of clades: $|\mathcal{C}|$,

with softmax activation function in the last layer and categorical crossentropy as loss function, since we want to assign only one clade to each DNA sequence.

Model evaluation

To assess the performance of our trained model, we perform a classification evaluation of the predictions and also a clustering evaluation for the embeddings in order to evaluate the class separability. The reported metrics are based on a Repeated 5-fold Cross Validation.

Classification metrics

We report global (accuracy and Matthews correlation coefficient) and class specific metrics (precision, recall, and F1-score) for the trained models.

Class specific metrics

Given a clade c, the correct predictions of the model can be compared to all the sequences with ground truth c (recall), and to all the sequences predicted by the model into the clade c (precision).

Formally, given a clade c, the positive class P consists of the set of genome sequences that are assigned to c, while all other genome sequences are the negative class N. Consequently, the true positive consist of the sequences that originate from the clade c and have been assigned to c, the false positive consist of the sequences that do not originate from the clade c and have been assigned to c, the false negative consist of the sequences that originate from the clade c and have not been assigned to c. The precision and recall are computed as follows:

$$precision = \frac{TP}{TP + FP}, \quad recall = \frac{TP}{TP + FN} \tag{3}$$

We also report the f1-score, defined as,

$$f1 - score = 2\frac{precision \times recall}{precision + recall} \tag{4}$$

Global model metrics

Given a classification problem on S samples and N classes, the corresponding confusion matrix $C = (c_{ij}), i, j \in [1, N]$ is a square matrix where each entry $c_{i,j}$ is the number of elements that belong to the true class i and were classified in the class j, and the sum of the entries in C is exactly S.

i. The **Accuracy** of the model is defined as the proportion of the corrected classified samples over the total number of samples, this value ranges between 0 and 1, where 0 means that all samples were erroneously classified, while a value of 1 means a perfect classification. It can be defined in terms of the entries of the confusion matrix as follows:

$$acc = \frac{\sum_{k=1}^{N} c_{kk}}{S} \tag{5}$$

ii. The **Matthews Correlation Coefficient** (MCC), proposed in [27] as a binary classification metric, was generalized to the multi-class case in 2004 [41], and it can be defined in terms of the confusion matrix as follows (see [42] for details):

$$MCC = \frac{cp \times S - \sum_{k=1}^{N} p_k \times t_k}{\sqrt{(S^2 - \sum_{k=1}^{N} p_k^2) \times (S^2 - \sum k = 1^N t_k^2)}} \tag{6}$$

where $cp = \sum_{k=1}^{N} c_{kk}$ is the total number of samples correctly predicted, $t_k = \sum_{i=1}^{N} c_{ik}$ is the number of times class k was truly occurred, and $p_k = \sum_{j=1}^{N} c_{kj}$ is the number of times class k was predicted. MCC lives in the range $[-1, 1]$, where 1 is perfect classification, −1 is the opposite, and 0 means that the confusion matrix is all zeros but for one single column, or when all entries are equal $c_{ij} = K \in \mathbb{N}$ [42].

Clustering measures

In order to assess the quality of the class separability given by the CNN, we evaluate the embeddings of the last layer (the one used to perform the classification) in the network with three clustering evaluation measures. These embeddings are the output from the final layer of the network for each FCGR.

i. **Silhouette Coefficient** [28] Given an embedding v belonging to a cluster A, the silhouette coefficient $s(v)$ of v compares the mean intra-cluster distance in A (a) with the mean nearest-cluster distance for v (b), that is, the closest cluster to v different from A.

$$s(v) = \frac{a - b}{\max\{a, b\}} \tag{7}$$

where $a = \frac{1}{|A|} \sum_{w \in A, w \neq v}^{|A|} d(v, w)$ and $b = \min_{B \neq A} \frac{1}{|B|} \sum_{w \in B}^{|B|, v \in A} d(v, w)$.
The value of $s(v)$ ranges between −1 (wrongly assigned) and 1 (perfect separability). For a cluster A, the mean silhouette coefficient of A is computed as the average of $s(v)$ over all embeddings $v \in A$.

ii. **Calinski-Harabasz Score** [29] Given a set of embeddings E of size n_E that has been clustered into k clusters, the Calinski-Harabasz Score s, also known as the Variance Ratio Criterion, is defined as the ratio of the between-clusters dispersion and the inter-cluster dispersion for all clusters (the dispersion of a group of n points is measured by the sum of the squared distances of the points from their centroid).

$$s = \frac{tr(B_k)}{tr(W_k)} \frac{n_E - k}{k - 1} \tag{8}$$

where $tr(B_k)$ is the trace of the between-cluster dispersion matrix and $tr(W_k)$ is the trace (the sum of all elements in the diagonal of W_k) of the within-cluster dispersion matrix, defined as follow:

$$W_k = \sum_{q=1}^{k} \sum_{v \in C_q} (v - c_q)(v - c_q)^T \tag{9}$$

$$B_k = \sum_{q=1}^{k} n_q (c_q - c_E)(c_q - c_E)^T \tag{10}$$

where C_q is the set of embeddings in the cluster q, c_q is the centroid of the cluster q, c_E is the centroid of E and $n_q = |C_q|$. The higher the score s means that the clusters are dense and well separated.

iii. **Generalized Discrimination Value (GDV)** [30]
Given a set of N D-dimensional embeddings $\{x_1, \ldots, x_N\}$, with $x_n = (x_{n,1}, \ldots, x_{n,D})$ and a set of L classes $\{C_1, \ldots, C_L\}$, where each x_n is assigned to one of the L distinct classes. Consider their z-scored points $(s_1, \ldots, s_N)$, with $s_i = (s_{i,1}, \ldots, s_{i,D})$, where $s_{n,d} = \frac{1}{2} \frac{x_{n,d} - \mu_d}{\sigma_d}$. Here $\mu_d = \frac{1}{N} \sum_{n=1}^{N} x_{n,d}$

denotes the mean, and $\sigma_d = \sqrt{\frac{1}{N}\sum_{n=1}^{N}(x_{n,d} - \mu_d)^2}$ the standard deviation of dimension d. Using the re-scaled data points $s_n = (s_{n,1}, \dots, s_{n,D})$, the Generalized Discrimination Value Δ is calculated from the mean intra-class and inter-class distances as follows:

$$\Delta = \frac{1}{\sqrt{D}}\left[\frac{1}{L}\sum_{l=1}^{L} d_{intra}(C_l) - \frac{2}{L(L-1)}\sum_{l=1}^{L-1}\sum_{m=l+1}^{L} d_{inter}(C_l, C_m)\right] \tag{11}$$

where the mean intra-class for each class C_l is defined as

$$d_{intra}(C_l) = \frac{2}{N_l(N_l-1)}\sum_{i=1}^{N_l-1}\sum_{j=i+1}^{N_l} d(s_i^{(l)}, s_j^{(l)}) \tag{12}$$

and the mean inter-class for each pair of classes C_l and C_m is defined as follows,

$$d_{inter}(C_l, C_m) = \frac{1}{\sqrt{D}}\left[\frac{1}{N_l N_m}\sum_{i=1}^{N_l}\sum_{j=1}^{N_m} d(s_i^{(l)}, s_j^{(l)})\right] \tag{13}$$

here N_k correspond to the number of points in class k, and $s_i^{(k)}$ is the ith point of class k. The quantity $d(a, b)$ is the distance between a and b, for our case, we considered the Euclidean distance. The value Δ range between −1 (perfect separability) and 0 (wrongly assigned),

Feature importance

After the model is trained, we can perform feature importance methods (also known as pixel attribution in case of images) to analyze the impact of each element of the FCGR in our prediction. We selected Saliency Maps [31] and Shap Values [32]. Saliency Maps calculate the gradient of the loss function for a specific desired class with respect to the input (FCGR) elements, the gradients are rescaled to $[0, 1]$, where elements with values closer to 1 represent the more influential features for the input FCGR over the predicted class. Shap (Shapley Additive Explanations) Values is a game theoretic approach to explain the output of any machine learning model. It aims to explain the influence of each feature compared to the average model output over the dataset the model was trained on, it outputs positive and negative values, where positive values push the prediction higher, and negative values push the prediction lower. Using the most relevant features from both methods over the FCGR, we aim to identify the most relevant k-mers for the classification of each clade.

Using these methods we aim to analyze the most relevant k-mers for the classification of each clade in the trained models.

Availability of source code and requirements

- Project name: Classification of SARS-CoV-2 genome sequence with CGR and CNN
- Project home page: https://github.com/AlgoLab/CouGaR-g
- Operating system(s): e.g. Platform independent
- Programming language: Python 3.10.5
- Other requirements: Python 3.10+, tensorflow 2.10.0, scikit-learn 1.1.2, tqdm 4.63.0, pandas 1.5.0, biopython 1.79, Pillow 9.0.1, matplotlib 3.5.1, shap 0.41.0, opencv-python 4.6.0.66
- License: GNU GPL

Availability of supporting data and materials

The list of FASTA sequences and metadata can be downloaded from https://www.gisaid.org/ after creating an account and accepting the *Terms of Use*. The data used in this study was downloaded on April 04, 2022. Trained models and results of our experiments can be downloaded from https://doi.org/10.5281/zenodo.7185290

A web app version of CouGaR-g with all the trained models is available online at https://huggingface.co/spaces/BIASLab/sars-cov-2-classification-fcgr

Declarations

List of abbreviations

- CNN: Convolutional Neural Networks
- CGR: Chaos Game Representation
- ENA: European Nucleotide Archive
- FCGR: Frequency matrix of Chaos Game Representation
- GDV: Generalized Discrimination Value
- SVM: Support Vector Machine

Consent for publication

Not applicable.

Competing Interests

The authors declare that they have no competing interests.

Funding

This project has received funding from the European Union's Horizon 2020 Innovative Training Networks programme under the Marie Skłodowska-Curie grant agreement No. 956229.

This project has received funding from the European Union's Horizon 2020 Research and Innovation Staff Exchange programme under the Marie Skłodowska-Curie grant agreement No. 872539.

Author's Contributions

JAC and SA wrote the code, prepared the data and performed the experiments. JAC, SA, SC, PB and GDV devised the methods and analyzed the results. JAC, SC and GDV designed the experiments. All authors contributed to finalizing of the manuscript.

Acknowledgements

The authors would like to thank Yuri Pirola, Raffaella Rizzi, Luca Denti, Murray Patterson, and Sarwan Ali for many useful discussions on the topic.

References

1. Khare S, Gurry C, Freitas L, Schultz MB, Bach G, Diallo A, et al. GISAID's Role in Pandemic Response. China CDC Weekly 2021;3(49):1049–1051. https://weekly.chinacdc.cn//article/id/21792cdf-a54a-4a11-b6fe-68d50f817d91.
2. Hadfield J, Megill C, Bell SM, Huddleston J, Potter B, Callender C, et al. Nextstrain: real-time tracking of pathogen evolution. Bioinformatics 2018 Dec;34(23):4121–

4123. https://academic.oup.com/bioinformatics/article/34/23/4121/5001388, publisher: Oxford Academic.
3. Chapter 15 - Immunodeficiency. In: Mak TW, Saunders ME, Jett BD, editors. Primer to the Immune Response (Second Edition) Boston: Academic Cell; 2014.p. 377–421. https://www.sciencedirect.com/science/article/pii/B9780123852458000157.
4. Ali S, Bello B, Chourasia P, Punathil RT, Zhou Y, Patterson M. PWM2Vec: An Efficient Embedding Approach for Viral Host Specification from Coronavirus Spike Sequences. arXiv:220102273 [cs, q-bio] 2022 Jan;http://arxiv.org/abs/2201.02273, arXiv: 2201.02273.
5. Ali S, Patterson M. Spike2Vec: An Efficient and Scalable Embedding Approach for COVID-19 Spike Sequences. In: 2021 IEEE International Conference on Big Data (Big Data); 2021. p. 1533–1540.
6. Ali S, Sahoo B, Ullah N, Zelikovskiy A, Patterson M, Khan I. A k-mer Based Approach for SARS-CoV-2 Variant Identification. In: Wei Y, Li M, Skums P, Cai Z, editors. Bioinformatics Research and Applications, vol. 13064 Cham: Springer International Publishing; 2021.p. 153–164. https://link.springer.com/10.1007/978-3-030-91415-8_14, series Title: Lecture Notes in Computer Science.
7. Jeffrey HJ. Chaos game representation of gene structure. Nucleic Acids Res 1990 Apr;18(8):2163–2170.
8. Almeida JS, Carriço JA, Maretzek A, Noble PA, Fletcher M. Analysis of genomic sequences by Chaos Game Representation. Bioinformatics 2001 May;17(5):429–437.
9. Deschavanne PJ, Giron A, Vilain J, Fagot G, Fertil B. Genomic signature: characterization and classification of species assessed by chaos game representation of sequences. Mol Biol Evol 1999 Oct;16(10):1391–1399.
10. Wang Y, Hill K, Singh S, Kari L. The spectrum of genomic signatures: from dinucleotides to chaos game representation. Gene 2005;346:173–185. https://www.sciencedirect.com/science/article/pii/S0378111904006481.
11. Löchel HF, Eger D, Sperlea T, Heider D. Deep learning on chaos game representation for proteins. Bioinformatics 2019 06;36(1):272–279. https://doi.org/10.1093/bioinformatics/btz493.
12. Dick K, Green JR. Chaos Game Representations amp; Deep Learning for Proteome-Wide Protein Prediction. In: 2020 IEEE 20th International Conference on Bioinformatics and Bioengineering (BIBE); 2020. p. 115–121.
13. Ren Y, Chakraborty T, Doijad S, Falgenhauer L, Falgenhauer J, Goesmann A, et al. Prediction of antimicrobial resistance based on whole-genome sequencing and machine learning. Bioinformatics 2021 10;38(2):325–334. https://doi.org/10.1093/bioinformatics/btab681.
14. Millán Arias P, Alipour F, Hill KA, Kari L. DeLUCS: Deep learning for unsupervised clustering of DNA sequences. PLOS ONE 2022 01;17(1):1–25. https://doi.org/10.1371/journal.pone.0261531.
15. Löchel HF, Heider D. Chaos game representation and its applications in bioinformatics. Computational and Structural Biotechnology Journal 2021;19:6263–6271.
16. Singer J, Gifford R, Cotten M, Robertson D. CoV-GLUE: a web application for tracking SARS-CoV-2 genomic variation; 2020.
17. Randhawa GS, Soltysiak MP, El Roz H, de Souza CP, Hill KA, Kari L. Machine learning using intrinsic genomic signatures for rapid classification of novel pathogens: COVID-19 case study. Plos one 2020;15(4):e0232391.
18. Sengupta DC, Hill MD, Benton KR, Banerjee HN. Similarity Studies of Corona Viruses through Chaos Game Representation. Comput Mol Biosci 2020 Sep;10(3):61–72.
19. Touati R, Haddad-Boubaker S, Ferchichi I, Messaoudi I, Ouesleti AE, Triki H, et al. Comparative genomic signature representations of the emerging COVID-19 coronavirus and other coronaviruses: High identity and possible recombination between Bat and Pangolin coronaviruses. Genomics 2020;112(6):4189–4202. https://www.sciencedirect.com/science/article/pii/S0888754320306364.
20. LeCun Y, Boser B, Denker JS, Henderson D, Howard RE, Hubbard W, et al. Backpropagation applied to handwritten zip code recognition. Neural computation 1989;1(4):541–551.
21. Lecun Y, Haffner P, Bottou L, Bengio Y. Object Recognition with Gradient-Based Learning. In: Contour and Grouping in Computer Vision Springer; 1999. .
22. Krizhevsky A, Sutskever I, Hinton GE. Imagenet classification with deep convolutional neural networks. Advances in neural information processing systems 2012;25.
23. Rizzo R, Fiannaca A, La Rosa M, Urso A. Classification Experiments of DNA Sequences by Using a Deep Neural Network and Chaos Game Representation. In: Proceedings of the 17th International Conference on Computer Systems and Technologies 2016 CompSysTech '16, New York, NY, USA: Association for Computing Machinery; 2016. p. 222–228. https://doi.org/10.1145/2983468.2983489.
24. Safoury S, Hussein W. Enriched DNA Strands Classification Using CGR Images and Convolutional Neural Network. In: Proceedings of the 2019 8th International Conference on Bioinformatics and Biomedical Science ICBBS 2019, New York, NY, USA: Association for Computing Machinery; 2019. p. 87–92. https://doi.org/10.1145/3369166.3369176.
25. Cacciabue M, Aguilera P, Gismondi MI, Taboga O. Covidex: An ultrafast and accurate tool for SARS-CoV-2 subtyping. Infection, Genetics and Evolution 2022;99:105261.
26. He K, Zhang X, Ren S, Sun J. Deep residual learning for image recognition. In: Proceedings of the IEEE conference on computer vision and pattern recognition; 2016. p. 770–778.
27. Matthews BW. Comparison of the predicted and observed secondary structure of T4 phage lysozyme. Biochimica et Biophysica Acta (BBA)-Protein Structure 1975;405(2):442–451.
28. Rousseeuw PJ. Silhouettes: A graphical aid to the interpretation and validation of cluster analysis. Journal of Computational and Applied Mathematics 1987;20:53–65. https://www.sciencedirect.com/science/article/pii/0377042787901257.
29. Caliński T, Harabasz J. A dendrite method for cluster analysis. Communications in Statistics-theory and Methods 1974;3(1):1–27.
30. Schilling A, Maier A, Gerum R, Metzner C, Krauss P. Quantifying the separability of data classes in neural networks. Neural Networks 2021;139:278–293. https://www.sciencedirect.com/science/article/pii/S0893608021001234.
31. Simonyan K, Vedaldi A, Zisserman A. Deep inside convolutional networks: Visualising image classification models and saliency maps. In: In Workshop at International Conference on Learning Representations Citeseer; 2014. .
32. Lundberg SM, Lee SI. A Unified Approach to Interpreting Model Predictions. In: Guyon I, Luxburg UV, Bengio S, Wallach H, Fergus R, Vishwanathan S, et al., editors. Advances in Neural Information Processing Systems 30 Curran Associates, Inc.; 2017.p. 4765–4774. http://papers.nips.cc/paper/7062-a-unified-approach-to-interpreting-model-predictions.pdf.
33. Abadi M, Agarwal A, Barham P, Brevdo E, Chen Z, Citro C, et al., TensorFlow: Large-Scale Machine Learning on Het-

erogeneous Systems; 2015. https://www.tensorflow.org/, software available from tensorflow.org.

34. Pedregosa F, Varoquaux G, Gramfort A, Michel V, Thirion B, Grisel O, et al. Scikit-learn: Machine learning in Python. Journal of machine learning research 2011;12(Oct):2825–2830.
35. Kingma DP, Ba J, Adam: A Method for Stochastic Optimization; 2017.
36. Ribeiro MT, Singh S, Guestrin C. " Why should i trust you?" Explaining the predictions of any classifier. In: Proceedings of the 22nd ACM SIGKDD international conference on knowledge discovery and data mining; 2016. p. 1135–1144.
37. Selvaraju RR, Das A, Vedantam R, Cogswell M, Parikh D, Batra D. Grad-CAM: Why did you say that? Visual Explanations from Deep Networks via Gradient-based Localization. CoRR 2016;abs/1610.02391. http://arxiv.org/abs/1610.02391.
38. Shrikumar A, Greenside P, Kundaje A. Learning important features through propagating activation differences. In: International conference on machine learning PMLR; 2017. p. 3145–3153.
39. Kokot M, Długosz M, Deorowicz S. KMC 3: counting and manipulating k-mer statistics. Bioinformatics 2017;33(17):2759–2761.
40. He K, Zhang X, Ren S, Sun J. Deep Residual Learning for Image Recognition. CoRR 2015;abs/1512.03385. http://arxiv.org/abs/1512.03385.
41. Gorodkin J. Comparing two K-category assignments by a K-category correlation coefficient. Computational biology and chemistry 2004;28(5-6):367–374.
42. Jurman G, Furlanello C. A unifying view for performance measures in multi-class prediction. arXiv preprint arXiv:10082908 2010;.

Manuscript

Click here to access/download;Manuscript;reviewer_comments.pdf

Responses to Reviewers
"Accurate and Fast Clade Assignment via Deep Learning and Frequency Chaos Game Representation"

We would like to thank the anonymous reviewers for their detailed and thoughtful comments. We believe that they have improved this article.

To help readability, we report the text of the reviewers into a box, while our responses are in plain text.

Review 1

> **Reviewer:** The paper is well written, and the objectives are clear. The study is a very nice application of CGR in bioinformatics and shows the excellent performance of CGR-encoded data in combination with deep learning. I have a few things that should be addressed in a minor revision:

Major comments:

> **Reviewer:** Some very important studies have not been addressed in the related work part, e.g., in Touati et al. (pubmed:32645523) and Sengupta et al. (pubmed:32953249), the authors compared SARS-CoV2 with other coronaviruses based on CGR, or we (pubmed:34613360) used CGR in combination with deep learning for resistance predictions in E. coli.

We thank the reviewer for the suggestion and we made sure to include them in the Introduction section. "Furthermore, an early approach to construct phylogenetics trees within SARS-CoV-2 strains and closely related species was proposed in [14] using FCGR as embedding for a Hierarchical Agglomerative Clustering. In a similar fashion, FCGR was explored along with other techniques as embedding for the identification of homologies between different known and emerging viruses in [15]."

> **Reviewer:** To me, it is unclear how accuracy was used in the model. Is it one class (i.e., clade) versus all others? If yes, accuracy might be misleading because of the high class imbalance. In such high class imbalances, MCC has been shown to be more suitable.

We thank the reviewer for this questions, and we clarify this in the manuscript by

including the MCC metric in our experiments, and by defining both accuracy and MCC in the Methods section.

- In subsection Model Evaluation - Classification metrics: *We report global (accuracy and Matthews correlation coefficient) and class specific metrics (precision, recall, and F1-score) for the trained models.*
- In subsection Model Evaluation - Global model metrics: *The Accuracy of the model is defined as the proportion of the corrected classified samples over the total number of samples, this value ranges between 0 and 1, where 0 means that all samples were erroneously classified, while a value of 1 means a perfect classification. The Matthews Correlation Coefficient (MCC), proposed in [40] as a binary classification metric, was generalized to the multi-class case in 2004 [41], and it can be defined in terms of the confusion matrix...*

Reviewer: "The undersampled dataset was randomly split into train...". Why did you undersample? To balance the data, which would make sense to use accuracy as a metric but discard a lot of valuable data. What about oversampling?

We thank the reviewer for this question, which allow us to clarify our approach to compensate the unbalanced dataset by using a weighted loss function for the training step. This was included in the Methods section.

- Third paragraph *Due to the huge amount of data available for most of the clades, we undersampled at most 20.000 sequences per clade to perform our experiments, nevertheless, for some clades only a portion of it was available (see table representativity). In order to overcome the unbalance in the undersampled dataset, we decided to use a weighted binary cross-entropy loss function (instead of oversampling the underrepresented classes), where the cost associated with a class c is inversely proportional to its representativity in the training set.*

Reviewer: Comparison with other tools: I wonder whether the good performance of your model is the result of deep learning or the CGR encoding. Please also provide the results for another ML model (besides SVM, e.g., random forests) to compare to, e.g., Covidex.

The proposed pipeline we describe in this paper uses a CNN to make use of the 2-dimensional structure given by the FCGR. Using traditional machine learning algorithms (SVM, Random Forest, Regression, Naive Bayes, K-Nearest Neighbors, etc) implies avoiding the use of the 2-dimensional structure, and break it down as a raw vector of frequencies of k-mers (according to Definition 2). We believe that both FCGR and Deep Learning cannot be evaluated separately, since (to the best of our knowledge) models that can receive as input the FCGR without disrupting the order of their elements fall into the field of Deep Learning, like for example Vision Transformers. However, since Covidex is a Random Forest based model, we do present results against other ML models (Covidex, a Random Forest based model), but as we emphasize, this approach

cannot use the FCGR.

We made clear this point in the second paragraph of *Comparison with the literature* section:

The main difference between both approaches is the model behind it, while Covidex uses Random Forest to perform the classification, we take advantage of the CNNs and use a 2-dimensional input, the FCGR. Notice that using the FCGR with any other classical Machine Learning method implies to convert the FCGR into a vector, and hence, the loss of the 2-dimensional structure.

Review 2

Reviewer: The authors propose a classification experiment based on Frequency Chaos Game Representation and deep learning. They used the outstanding performances of a ResNet network as an image classification tool and the FCGR method that represent a genome sequence as an image.

The work seems good, although some major points should be clarified.

Major Comments

Reviewer: First, whether the performance index values came from a 5-fold validation procedure (5 because they said the split was 80-10-10) or a one-shot experiment is unclear.

In the previuos work all results were from a one shot experiment. Thanks to the observation of the reviewer, we overcome this comment by running again our experiments using a 5-fold validation procedure. Results for the classification metrics of the model (accuracy and MCC, Precision, Recall and F1score) for each model and Clade can be seen in Table 3 and Table 4, while results for clustering metrics (Silhouette coefficient, Calinski-Harabasz and Generalized Discrimination Value) can be seen in Table 6. All the metrics are reported by $\mu \pm \sigma$, where μ and σ correspond to the mean and standard deviation of the metrics in the 5-fold validation experiment, respectively.

Reviewer: Second, the part that involves the frequent k-mers and the SVM should be better explained. The authors should clarify what the meaning of this comparison is.

We thank the reviewer for this comment, and we clarify the purpose this experimentation in the first paragraph of *Relevant k-mers for the clasification of each clade* section.

The purpose of this experiment is to study if a set of the most relevant k-mers (based on feature importance methods) are informative enough to a SVM to perform similarly than the trained CNNs (that uses FCGR as input, and hence all the 4^k *possible k-mers)*

Reviewer: Another point to clarify is the quality of the sequences used; the authors worked on complete sequences, but, as far as I know, in the real world virus sequences are noisy data, and authors should discuss this point.

We downloaded sequences from GISAID coming only from high coverage and high quality sequencing data. We expect those data to be much less noisy than average viral data (which are noisy, as the reviewer points out).

Minor Comments

Reviewer:

- Authors said that a sequence is a string $s \in \{A, C, G, T, N\}^*$, so they should explain the procedure used in Definition 2, where only 4 symbols seem to be used. If they discard the N, or consider 4 k-mers (consider that N means "any symbol") they should say it clearly.
- Figure 1 and 2 report two different quantities but say the same thing; maybe one of them can be omitted.
- Authors should add some details about the training time of the network.

For the first point we included two paragraphs, one mentioning that sequences with missing bases can be problematic, and the other one mentioning how to overcome this situation by using definition 2, by simply excluding portions of the sequences with the N character.

- Background, previous paragraph to Definition 2: *Missing bases can be problematic to encode, since the $g(\cdot)$ function is not defined in that case, we used the notion of frequency matrix CGR [9, 8], which has the added benefit of allowing us to manage k-mers instead of strings of arbitrary length.*
- *Note that the FCGR is defined for a DNA sequence with unknown nucleotide, denoted by N — where k-mers with an N are simply excluded in the counting process— while the CGR encoding is well-defined only when all nucleotides are known. To explicitly mention the dimension of the FCGR, we will refer to this as the k-th order FCGR.*

For the second point we agree with the reviewer and only show one of them, the one related to the loss.

For the third point, in Table 2 was included a new column reporting the average time per epoch that takes each model for the different values of k-mer considered.

We thank the reviewer for the suggestions and we fixed the aforementioned issues.

Reviewer: A final suggestion: probably it will be interesting to use the same deep network with transfer learning (the whole network or just the first sections) to evaluate the gain with ad-hoc training and the different training time.

We perform some experiments at the beginning of our exploration with transfer learning, but they were not reported in the previous manuscript. In this case we included a paragraph in the Discussion session:

- Second paragraph: *We decided to exclude transfer learning from our experiments after trying this approach without success on 8-mers. For this trial we used pre-trained weights from the Imagenet dataset using Resnet50 architecture, where the backbone weights were frozen, and three dense layers were included at the top of it for the classification*

Manuscript

Click here to access/download;Manuscript;main.pdf

GigaScience, 2017, 1–10

doi: xx.xxxx/xxxx
Manuscript in Preparation
Paper

PAPER

Accurate and Fast Clade Assignment via Deep Learning and Frequency Chaos Game Representation

Jorge Avila Cartes[1], Santosh Anand[1], Simone Ciccolella[1], Paola Bonizzoni[1] and Gianluca Della Vedova[1,*]

[1]Department of Computer Science, Systems and Communications. University of Milano–Bicocca, Italy

*gianluca.dellavedova@unimib.it

Abstract

Background: Since the beginning of the COVID-19 pandemic there has been an explosion of sequencing of the SARS-CoV-2 virus, making it the most widely sequenced virus in the history. Several databases and tools have been created to keep track of genome sequences and variants of the virus, most notably the GISAID platform hosts millions of complete genome sequences, and it is continuously expanding every day. A challenging task is the development of fast and accurate tools that are able to distinguish between the different SARS-CoV-2 variants and assign them to a clade.
Results: In this paper, we leverage the Frequency Chaos Game Representation (FCGR) and Convolutional Neural Networks (CNNs) to develop an original method that learns how to classify genome sequences that we implement into CouGaR-g, a tool for the clade assignment problem on SARS-CoV-2 sequences. On a testing subset of the GISAID, CouGaR-g achieves an 96.29% overall accuracy, while a similar tool, Covidex, obtained a 77, 12% overall accuracy. As far as we know, our method is the first using Deep Learning and FCGR for intra-species classification. Furthermore, by using some feature importance methods CouGaR-g allows to identify k-mers that matches SARS-CoV-2 marker variants.
Conclusions: By combining FCGR and CNNs, we develop a method that achieves a better accuracy than Covidex (which is based on Random Forest) for clade assignment of SARS-CoV-2 genome sequences, also thanks to our training on a much larger dataset, with comparable running times. Our method implemented in CouGaR-g is able to detect k-mers that capture relevant biological information that distinguishes the clades, known as marker variants.
Availability: The trained models can be tested online providing a FASTA file (with one or multiple sequences) at `https://huggingface.co/spaces/BIASLab/sars-cov-2-classification-fcgr`. CouGaR-g is also available at `https://github.com/AlgoLab/CouGaR-g` under the GPL.

Key words: Chaos Game Representation, Convolutional Neural Networks, Classification of genome sequences, SARS-CoV-2, GISAID clades, k-mer frequency, deep learning.

Introduction

The global coordination in combating the COVID-19 pandemic has led to the sequencing of one of the largest amount of viral genomic data ever produced. All this data is stored in publicly available archives, such as the European Nucleotide Archive (ENA) and GISAID [1], currently having more than 9.6 million sequenced genomes, classified in *variants*, *clades*, and *lineages*.

The SARS-CoV-2 virus has evolved since its discovery, and the currently available phylogenies describing its evolutionary history [2] show more than 2000 different genomes, divided into lineages. Since the phylogeny is fairly stable and the main (existing) *lineages*, i.e. the lines of descent, have been identified, a natural and interesting problem is to quickly find, given a sequence, the *clade* to which it belongs, i.e. a group of descendants sharing a common ancestor [3]. Fast and efficient solutions to the clade assignment problem would help in tracking current and evolving strains and it is crucial for the surveillance of the pathogen. This classification problem has been attacked with machine learning

Compiled on: October 17, 2022.
Draft manuscript prepared by the author.

approaches [4, 5, 6] using the Spike protein amino acid sequence to drive the classification step.

In this paper we propose a method for classifying SARS-CoV-2 genome sequences based on Chaos Game Representation (CGR) [7]: a deterministic bidimensional representation of a DNA sequence, also called CGR encoding, that can be easily obtained from the genome sequences. The CGR encoding of a sequence has two fundamental properties: it is deterministic, that is there is a unique CGR encoding of each sequence, and reversible, hence the original sequence can be recovered from its representation [8].

A strongly related approach, known as Frequency matrix of Chaos Game Representation (FCGR) [9, 8], starts from the k-mers (the substring of length k) of the string we want to represent resulting in the the notion of k-th order FCGR [10]. The *k-th order FGCR* of a sequence s is a $2^k \times 2^k$ matrix whose elements are the number of occurrences, i.e. the frequencies, of each k-mer in s, where each frequency is stored in the specific and distinct position for each k-mer. Note that the matrix shape depends on the fact that the sequence s is on a 4-symbol alphabet. In essence, the FCGR is an alternative ordering of the histogram for all the k-mers (for a fixed integer k). Deep Learning and FCGR have been used to evaluate the drug resistance for protein sequences of HIV [11]; for multi-class classification task to identify the source organism for a given protein [12] — in this case the FCGR has been extended to encode sequences in the protein alphabet — and to predict antimicrobial resistance of different drugs in *E. Coli* [13]. The FCGR has also been used for unsupervised clustering of DNA sequences of several species [14] by using dense neural networks, where the input of these networks must be a 1-dimensional vector. In this case, the 2-dimensional FCGR representation of the sequences must be flattened and cannot be fully exploited. For an extensive review on CGR and its applications in bioinformatics, we refer the reader to [15].

Subtyping Sars-Cov-2 sequences has been addressed in the literature with bioinformatics pipelines that require the alignment to a reference genome [2] [16], and also with machine learning approaches aiming to skip the alignment step [17]. Furthermore, an early approach to construct phylogenetics trees within SARS-CoV-2 strains and closely related species was proposed in [18] using FCGR as embedding for a Hierarchical Agglomerative Clustering. In a similar fashion, FCGR was explored along with other techniques as embedding for the identification of homologies between different known and emerging viruses in [19]. Convolutional Neural Networks (CNNs) [20, 21], showed outstanding results in the well-known Imagenet classification problem [22]. To the best of our knowledge, only two works have used CNNs and FCGR for the classification of DNA sequences. In [23] a simplification of the network reported in [21] was used to classify different taxonomic categories with a dataset of 3,000 sequences (1200–1400 long). A comparison with Support Vector Machines (SVM), showed that CNNs improve over SVM when using a fragment (500bp) of the sequences. In [24], a CNN was proposed for the classification of a dataset of ≈ 660 sequences from eleven phylogenetic families reporting a test accuracy of 87%.

In this paper we leverage the FCGR representation of genomic sequences and CNN power to perform intra-species classification of viral DNA genome sequences, using SARS-CoV2 as our case of study and GISAID clades as our labels. Observe that in this problem the CNN classifies a dataset that is at least two order of magnitude larger than the one considered in the above mentioned papers. Another work that has tackled the clade assignment problem is Covidex [25], a web app tool based on Random Forest and k-mer frequencies: to the best of our knowledge this is the most recent work facing our problem. Notice that almost the entire phylogenetics literature deals with inter-species classification, where the distance between possible cluster centroids is larger, hence the classification problem is easier. We propose to use a residual neural network [26] (ResNet50) for the classification of DNA sequences into 11 GISAID clades, using a dataset of two orders of magnitude larger (153K sequences for training) than those analyzed in the above cited works (about 3000 sequences in [23]).

Classification metrics (accuracy, Matthews Correlation Coefficient [27], precision, recall and f1-score) and analysis of the separability of the embeddings generated by the classification layer (Silhouette Coefficient [28], Calinski-Harabasz Score [29], and Generalized Discrimination Value (GDV) [30]) are analyzed for each model. Using the fact that each feature in the FCGR is uniquely related to a k-mer, we aim to analyze if the most relevant k-mers identified by feature importance methods (Saliency Maps [31] and Shap Values [32]) are related to mutations defining each clade.

We trained four models, one for each value of $k \in \{6, 7, 8\}$. All models performed very similarly, with $k = 8$ being the best one, achieving an overall accuracy of 96.22% in the test set, and the best classification metrics (0.948 for Silhouette Coefficient, 174, 736.1 for Calinski-Harabasz and −0.718 for GDV). Three clades (O, GR and GRY) reported the lowest f1-score for all the trained models. Since GR is a close ancestor of GRY and these two clades share many mutations, they are confused with each other. For clade O, mispredictions are among most of the clades.

Using the 20 most relevant k-mers identified by Saliency Maps, we were able to achieve a similar performance than our CNNs models using SVM for $k \in \{6, 7, 8\}$. Finally, to access the performance of our models w.r.t. other approaches, we compare our results with Covidex [25] the only recent tool that we found in the literature solving the clade assignment problem. Our results show that our models outperform Covidex in all clades and reported metrics (accuracy, precision, recall and f1-score).

Background

The Chaos Game Representation for encoding DNA/RNA sequences is formally defined as:

Definition 1 (Chaos Game Representation (CGR)) *Let $s = s_1 \dots s_n \in \{A, C, G, T\}^*$ be a sequence. Then the CGR encoding of the sequence s is the bidimensional representation of the ordered pair (x_n, y_n) which is defined iteratively as*

$$(x_i, y_i) = \frac{1}{2}\Big((x_{i-1}, y_{i-1}) + g(s_i)\Big), \text{ if } i \geq 1 \quad (1)$$

where $(x_0, y_0) = (0, 0)$ and,

$$g(s_i) = \begin{cases} (1,1) & s_i = A \\ (-1,1) & s_i = C \\ (-1,-1) & s_i = G \\ (1,-1) & s_i = T \end{cases} \quad (2)$$

Note that each point (x_i, y_i) obtained with the above encoding represents the i-long prefix of the sequence s. Also, all the CGR encodings are points inside the square with vertices given by the values of the function g. In particular, the encoding of all prefixes that shares the last character will be placed in the same quadrant, all prefixes that shares the two last characters, will be placed in the same sub-quadrant, and so on. This property results in a fractal structure of the representation.

Missing bases can be problematic to encode, since the $g(\cdot)$ function is not defined in that case, we used the notion of frequence matrix CGR [9, 8], which has the added benefit of allowing us to manage k-mers instead of strings of arbitrary length.

Definition 2 (Frequency matrix of Chaos Game Representation) *Let $s = s_1 \dots s_n \in \{A, C, G, T, N\}^*$ be a sequence, and let k be an integer. Then the frequency matrix of CGR, in short FCGR, of the sequence s is a*

$2^k \times 2^k$ *bidimensional matrix* $F = (a_{i,j}), 1 \le i, j \le 2^k, i, j \in \mathbb{N}$. *For each* k-*mer* $b \in \{A, C, G, T\}^k$, *we have an element* $a_{i,j}$ *in the matrix* F, *that is equal to the number of occurrences of* b *as a substring of* s. *Moreover, the position* (i, j) *of such element is computed as follows:*

$$i = 2^k - \lceil 2^{k-1}(x+1) \rceil + 1$$
$$j = \lceil 2^{k-1}(y+1) \rceil$$

where (x, y) *is the CGR encoding for the* k-*mer* b.

Note that the FCGR is defined for a DNA sequence with unknown nucleotide, denoted by N — where k-mers with an N are simply excluded in the counting process— while the *CGR* encoding is well-defined only when all nucleotides are known. To explicitly mention the dimension of the FCGR, we will refer to this as the k-th order FCGR.

Classification of viral sequences of DNA

We are given a phylogeny over the possible viral strains, partitioned into classes: each class c of such partition $\mathcal{C}$ is a *clade* of the tree. More precisely, a clade is a group of related organisms descended from a common ancestor [3], in other words a clade is a subtree of a phylogeny that consist of an ancestral lineage and all its descendants.

Given a genome sequence, that is a string $s \in \{A, C, G, T, N\}^*$, we determine the original clade in $\mathcal{C}$ from which the genome sequence is originated; however the genome sequence s might not have been previously observed. In any case the sequence will be assigned to a putative clade. To solve this problem, we propose a supervised learning model based on Convolutional Neural Networks (CNN) [21], using FCGR as inputs.

Data Description

The dataset for this experiment was downloaded from GISAID. By the time of our access to GISAID [1] there were around 10 million sequences.

In order to undersample the available data, we first dropped all the rows in the metadata without information in the columns `Virus name`, `Collection Date`, `Submission Date`, `clade`, `Host` and `Is complete?`, then we built a `fasta_id` identifier from the metadata as a concatenation of the columns `Virus name`, `Collection Date` and `Submission Date`.

For each clade, we randomly selected 20,000 sequences considering only those rows where the `Host` column has value "Human" — clades L, V, and S have less than 20,000 sequences available, in these cases all sequences have been selected.

As a result of the above procedure, we obtained 191,456 sequences among the 11 GISAID clades (S, L, G, V, GR, GH, GV, GK, GRY, O, and GRA) over the 12 availables, we excluded the clade GKA from our study since there were only 81 sequences reported in the metadata. The undersampled dataset was randomly split into train, validation and test sets in 80 : 10 : 10 proportion, preserving the same proportion of clades (labels) in each set. The distribution of the clades over the datasets is given in Table 1

Analyses

In this section we present the experimental setup, the dataset used to train and test each model, and clustering and classification metrics. We train one model for each $k \in \{6, 7, 8\}$ and we complement the study of the accuracy of each model (compared against Covidex [25]) with an analysis of the most relevant k-mers for the classification of each clade using Saliency Maps and Shap.

For this experiment we choose $k \in \{6, 7, 8\}$ and sequences from 11 GISAID clades: S, L, G, V, GR, GH, GV, GK, GRY, O and GRA.

Experimental setup

All experiments are conducted using a Intel(R) Core(TM) i5-10400 CPU @ 2.90GHz, x86_64, 32 GB RAM and a graphic card NVIDIA GeForce RTX 3060. The implementation is done in Python 3.10.5. Tensorflow 2.10.0 [33] was used for training the CNN and scikit-learn 1.1.12 [34] to compute classification metrics and clustering evaluation (except for Generalized Discrimination Value that was implemented). All code is available online for reproducibility [2].

Clade	Train	Val	Test	Total	Available
S	14,298	1,788	1,788	17,874	17,874
L	5,154	644	644	6,442	6,442
G	15,999	2,000	2,000	20,000	408,552
V	5,713	714	714	7,141	7,141
GR	16,000	2,000	2,000	20,000	625,662
GH	16,000	2,000	2,000	20,000	547,792
GV	16,000	2,000	2,000	20,000	182,248
GK	16,000	2,000	2,000	20,000	4,170,758
GRY	16,000	2,000	2,000	20,000	944,876
O	16,000	2,000	2,000	20,000	55,400
GRA	16,000	2,000	2,000	20,000	2,833,863
	153,164	19,146	19,146	191,456	9,800,608

Table 1. Distribution of the number of sequences selected for train, validation and test sets by each clade. The final dataset for the 11 clades was split in a 80 : 10 : 10 proportion for train, validation, and test sets.

Model training

Each model was set to be trained for 50 epochs with a batch size of 32 using Adam optimizer [35] with learning rate 0.001 (the default parameters in keras). The validation loss was monitored after each epoch to save the best trained weights, reducing the learning rate with a patience of 8 epochs and a factor of 0.1, and by an early stopping in case the metrics do not improve after 12 epochs.

We show the accuracy (average of the 5 Repeated fold cross validation) of the train and validation sets for $k = 8$ in Figure 1. For $k = 6$ and $k = 7$ the training is more unstable for the first epochs, but it behaves similar to $k = 8$ in the later epochs, i.e. training and validation metrics are similar.

The architecture used in this experiment is the same for all k (ResNet50 [26]), we only changed the input size. Originally, this architecture was designed for inputs of size $(224 \times 224 \times 3)$, which led us to the assumption that this architecture could be more suitable for $k = 8$. Notice that our sequences are $\approx 29,000bp$ long, which means that our input FCGR for $k = 8$ is very sparse, since from an n-long sequence we can count $n - k + 1$ k-mers, it means that (in the case where all k-mers are different) we have at most 29,000 k-mers, at least 55% of the elements of the FCGR are 0 for $k = 8$. In Table 2 a comparison of the number of features for each k and the training time per epoch in our experiments is detailed.

1 April 04, 2022. https://www.gisaid.org/

2 https://github.com/AlgoLab/CouGaR-g

k-mer	Dimensions	Features	Size (GiB)	Time per epoch (min)
6	(64,64)	4,096	6.6	4:05
7	(128,128)	16,384	24.1	8:21
8	(256,256)	65,536	94.2	24:50

Table 2. For each k, the dimension of the FCGR, its number of features (4^k), the amount of memory required to store the selected dataset of 191, 456 sequences as FCGR, and the average training time per epoch are reported in the table. The number of features and the space increase exponentially w.r.t k.

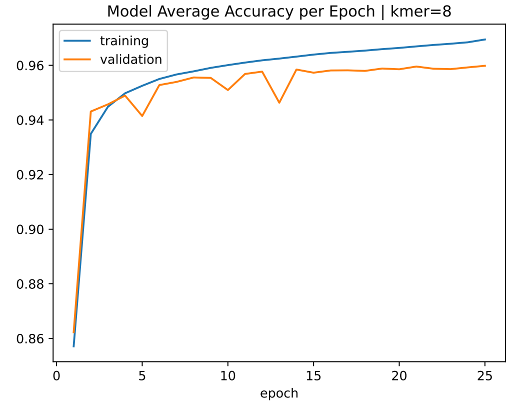

Figure 1. Average accuracy in the training and validation sets for our model with $k = 8$. The best model (final weights) is set as the one with the lowest validation loss, achieved at epochs 24 ± 5 for $k = 8$ (from a 5 RepeatedFold cross validation process). All models were trained for 50 epochs using an early stopping of 12 epochs based on the validation loss (hence, not all of them ran for 50 epochs).

k-mer	Accuracy	MCC
6	0.953714 ± 0.001589	0.948792 ± 0.001813
7	0.959856 ± 0.001740	0.955566 ± 0.001910
8	$\mathbf{0.962175 \pm 0.002829}$	$\mathbf{0.958211 \pm 0.003141}$

Table 3. Accuracy and Matthews Correlation Coefficient in the set for each of our models. Each metric ($\mu \pm \sigma$) is reported by its average (μ) and standard deviation (σ) from a 5 RepeatedFold cross validation process. For both, accuracy and MCC the model increases with the value of k. Going from k=6 to k=8 increases accuracy in 0.84%, and MCC in 0.94%. In **bold** the highest value of each metric.

Classification results

After each model is trained the precision and recall for the test set are computed for each clade using the best trained weights (lowest loss in the validation set), achieved at epochs 24 ± 5, 27 ± 8, and 20 ± 3 for $k = 6$, $k = 7$, and $k = 8$, respectively. In our case, we assign each sequence to the clade with highest score. Precision, recall and f1-score are shown in Table 4.

Precision and recall are very similar among all the trained models, with small improvements when k increases, 5 out of 11 clades have f1-score greater than 99% in our best model ($k = 8$). Most notable differences in the performance can be seen in clades GR and GRY, which present the lowest (and under 90%)reported recall and precision in each model, respectively. Moreover, from the confusion matrices (see Fig. 2) we can see that misclassified sequences that belong to clades GR and GRY, are confused between them, this can be explained since clade GRY is originated from clade GR. For the other clades, most of the misclassified sequences are predicted as (or belong to) clade G, that is the former one. Clade O exhibits the second lowest recall, where the misclassified sequences are assigned predominantly to clades G, GH, GK, and GRY.

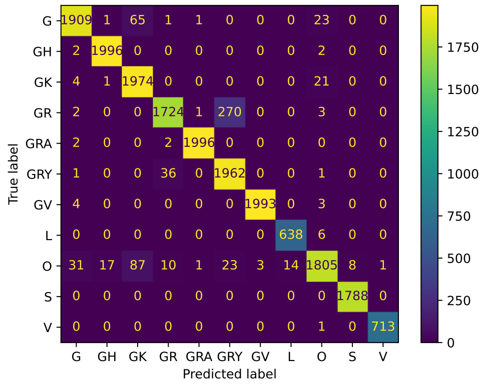

Figure 2. Confusion matrix for the test set for one of the trained models with $k = 8$ (from a 5 RepeatedFold cross validation process). All the models are able to correctly classify more than 98% of the sequences for all clades except for G, GR, GRY, and O. Most of the incorrectly classified sequences of GR and GRY are confused between them, which makes sense since they are evolutionary related. For the G clade, the incorrectly classified sequences are shared between clades GK and O. For the clade O, the incorrectly classified sequences are predominantly assigned to clades G, GH, GK, and GRY.

Comparison with the literature

We compare our results against Covidex [25], a tool that classify Sars-CoV-2 sequences into three nomenclatures: GISAID, Nextrain and Pango lineages. Using a different model for each task, all based on Random Forest and 6-mers as input. The reported accuracy are 97, 77%, 99, 52% and 96, 56% for GISAID, Nextstrain and Pango models, respectively. They also trained the models using 7-mers, but they claim that it only produced slightly better results in terms of accuracy but with more than doubling the computation time [25].

The input for Covidex is a vector with the normalized counting of the frequencies for all 4^k k-mers. Our input, the FCGR also considers all k-mers but in a bidimensional matrix. The main difference between both approaches is the model behind it, while Covidex uses Random Forest to perform the classification, we take advantage of the CNNs and use a 2-dimensional input, the FCGR. Notice that using the FCGR with any other classical Machine Learning method implies to convert the FCGR into a vector, and hence, the loss of the 2-dimensional structure.

Since our model is trained using GISAID clades, we only compare to those results. In Covidex, they used 10 clades: S, L, G, V, GR, GH, GV, GK, GRY and O. In our case, we included GRA since there were enough available sequences by the time of our experiments, but this is not considered in the comparison.

For Covidex, the model for the GISAID nomenclature was trained with 66, 126 sequences and tested on 13, 230. Since Covidex is made available as an user app for any SARS-Cov2 sequence, we used the app over our test dataset to compare the results. We tested Covidex on our test dataset of 17, 146 sequences (excluding the 2000 sequences from GRA clade). Achieving a 77, 12% of accuracy, more than a 18% lower than all our trained models and 20, 65% lower than their reported accuracy. The reported precision, recall and f1-score, as well as the test results over our selected dataset can be seen in Table 5. We found that the reported f1-score of Covidex is quite distant for the one we obtained in our test dataset for clades L (−8.4%), G (−15.8%), GR (−42.4%), GK (−10.9%), GRY (−19.3%) and O (−28.8%), while for clades S (−0.8%), V (−2.9%), GH (−2.5%) and GV (−0.9%), we can observe a decrement on the reported f1-score ranging from 0.8% to 2.9%. Our models (see Table 4) exhibit better performance than Covidex in all clades and metrics on our test set, with similar results only on clades S and GV. We did not perform an extensive comparison of the running times since both

k-mer	6			7			8		
Clade	Precision	Recall	F1score	Precision	Recall	F1score	Precision	Recall	F1score
S	99.4 ± 0.2	99.6 ± 0.2	99.5 ± 0.1	99.7 ± 0.1	99.6 ± 0.1	99.7 ± 0.1	99.8 ± 0.2	99.7 ± 0.3	**99.8 ± 0.1**
L	98.3 ± 0.4	97.7 ± 0.6	98.0 ± 0.3	98.7 ± 0.4	99.0 ± 0.2	**98.9 ± 0.2**	98.0 ± 0.4	99.5 ± 0.3	98.7 ± 0.2
G	95.8 ± 0.7	94.7 ± 0.8	95.2 ± 0.4	97.1 ± 0.4	95.1 ± 0.4	96.1 ± 0.3	97.3 ± 0.8	95.9 ± 0.7	**96.6 ± 0.1**
V	99.1 ± 0.4	99.2 ± 0.4	99.1 ± 0.2	99.5 ± 0.5	99.4 ± 0.3	99.4 ± 0.3	99.6 ± 0.5	99.6 ± 0.2	**99.6 ± 0.3**
GR	91.7 ± 2.0	85.9 ± 1.7	88.7 ± 0.4	92.4 ± 1.3	87.5 ± 1.4	89.8 ± 0.2	93.9 ± 2.3	86.6 ± 1.7	**90.1 ± 0.7**
GH	98.6 ± 0.3	99.5 ± 0.1	99.0 ± 0.2	98.9 ± 0.2	99.7 ± 0.1	**99.3 ± 0.1**	98.8 ± 0.2	99.8 ± 0.1	**99.3 ± 0.1**
GV	99.5 ± 0.3	99.6 ± 0.2	99.5 ± 0.1	99.7 ± 0.1	99.6 ± 0.1	99.7 ± 0.1	99.6 ± 0.1	99.8 ± 0.1	**99.7 ± 0.0**
GK	91.8 ± 0.4	97.6 ± 0.5	94.6 ± 0.2	92.2 ± 0.5	97.7 ± 0.2	94.9 ± 0.4	92.7 ± 0.2	98.1 ± 0.8	**95.3 ± 0.4**
GRY	86.4 ± 1.3	93.2 ± 2.4	89.7 ± 0.6	87.8 ± 1.1	93.9 ± 1.4	90.7 ± 0.3	87.1 ± 1.5	95.5 ± 2.0	**91.0 ± 0.7**
O	94.3 ± 0.8	86.8 ± 0.2	90.4 ± 0.4	95.0 ± 0.6	89.2 ± 0.8	92.1 ± 0.6	96.7 ± 0.4	88.8 ± 1.2	**92.6 ± 0.7**
GRA	99.7 ± 0.1	99.8 ± 0.1	99.8 ± 0.1	99.9 ± 0.1	99.7 ± 0.1	**99.8 ± 0.0**	99.8 ± 0.1	99.8 ± 0.1	99.8 ± 0.1

Table 4. Precision, recall, and f1-score. Each of our models is represented by length of the the k-mers used to generate the FCGR. Two clades, GR and GRY present deviations greater than 1% in their precision and recall for all values of k. In **bold** the highest F1score for each clade and k. Each metric ($\mu \pm \sigma$) is reported by its average (μ) and standard deviation (σ) from a 5 RepeatedFold cross validation process.

	Report			Test		
Clade	Prec.	Rec.	F1score	Prec.	Rec.	F1score
S	0.998	1	0.999	**0.988**	**0.995**	**0.991**
L	0.997	1	0.999	0.859	**0.979**	0.915
G	0.993	0.984	0.989	0.811	0.852	0.831
V	1	1	1	0.958	**0.985**	**0.971**
GR	0.945	0.915	0.930	0.379	0.760	0.506
GH	0.995	0.999	0.997	0.957	**0.987**	**0.972**
GV	0.996	0.999	0.997	**0.980**	**0.995**	**0.988**
GK	0.977	0.995	0.986	0.925	0.833	0.877
GRY	0.920	0.961	0.940	0.732	0.763	0.747
O	0.994	0.959	0.976	0.722	0.658	0.688

Table 5. Precision, recall, and f1-score for Covidex. The Report part is taken from the Supplementary material of [25]. The Test part has the precision, recall, and f1-score obtained by Covidex on our test set, restricted to the 10 clades (17,146 sequences) analyzed in [25]. We found significant differences between Covidex and our trained models in the Test metrics (see Table 4). In particular, the most notorious differences w.r.t f1-score, ranging from 8.4%–42.4% are found for clades L (−8.4%), G (−15.8%), GR (−42.4%), GK (−10.9%), GRY (−19.3%) and O (−28.8%), while for clades S (−0.8%), V (−2.9%), GH (−2.5%) and GV (−0.9%), we can observe a decrement on the reported f1-score ranging from 0.8%–2.9%. Metrics in **bold** in the Test part are those which **did not decrease** more than 3% w.r.t the reported metrics.

tools classify a genome sequence in less than a second (on $k = 8$, our tool took 0.15 seconds in average).

Clustering results

We evaluate the embeddings of the last layer of each trained model using the Silhouette Coefficient, Calinski-Harabasz score and Generalized Discrimination Value (GDV). These results are shown in Table 6. We can observe that the model for $k = 9$ is the best one among all metrics, however, all trained models exhibit a very similar separability based on Silhouette and GDV.

k-mer	Silhouette	Calinski-Harabasz	GDV
6	0.939 ± 0.007	145,879.214 ± 18,132.428	-0.712 ± 0.003
7	0.948 ± 0.003	163,926.767 ± 8,638.391	-0.717 ± 0.002
8	0.948 ± 0.006	174,736.086 ± 22,554.904	-0.718 ± 0.003

Table 6. Clustering metrics for our trained models. Each metric is computed using the output of each model and the predicted clade (that is, the clade that achieves the highest score by our model) in the test set. Each model is represented by the length of the k-mers used to generate the FCGR. For the Silhouette score, the closest to 1 the better. For the Calinski-Harabasz score, larger values are better. For the GDV score, the closest to -1, the better. All models exhibit comparable separability of the clusters. Each metric ($\mu \pm \sigma$) is reported by the average (μ) and standard deviation (σ) in the from a 5 RepeatedFold cross validation process.

Relevant k–mers for the classification of each clade.

The purpose of this experiment is to study if a set of the most relevant k-mers (based on feature importance methods) are informative enough to a SVM to perform similarly than the trained CNNs (that uses FCGR as input, and hence all the 4^k possible k-mers).

Using Saliency Maps and Shap Values, we can evaluate the contribution of each element of a FCGR in the classification, for each model. From each one of these feature attribution methods we can obtain an ordered list of all k-mers. For each clade, we use the centroid FCGR of all correctly classified sequences in the test set, then we use each centroid FCGR to identify the most relevant k-mers for each clade and then train a SVM using the N most relevant k-mers (for different values of $N \in \{1, 2, 3, 4, 5, 10, 15, 20, 25, 30, 35, 40, 45, 50\}$) and their respective frequencies as input.

The same training and test sets used for the CNNs were used for the SVM. The results of the accuracy in the test set for the different values of N are shown in Figures 6 and 5. We can observe that k-mers identified by Saliency Maps are more informative than those identified by Shap Values, since for $N = 20$, we obtain similar accuracy in the test set for $k = 6, 7, 8$ compared to CNN (96–97%), while in the case of Shap Values, this accuracy is only achieved by $k = 7$ with $N = 35$. Notice that using $N = 20$, we are considering a small number of all possible k-mers (0.49% for $k = 6$, 0.12% for $k = 7$ and 0.03% for $k = 8$).

Matching relevant k-mers to mutations.

Using the reference genome employed by GISAID (EPI_ISL_402124) [3] and the list of marker variants [4] for each GISAID clade with respect to this reference, we evaluated how many k-mers among the 50 chosen ones by Saliency Maps and Shap Values actually matched any of the reported marker variants. A summary is shown in Table 7.

The results shown that the most relevant k-mers selected using Saliency Maps match several of the reported marker variants (46

3 https://www.gisaid.org/resources/hcov-19-reference-sequence/
4 https://www.gisaid.org/resources/statements-clarifications/clade-and-lineage-nomenclature-aids-in-genomic-epidemiology-of-active-hcov-19-viruses/

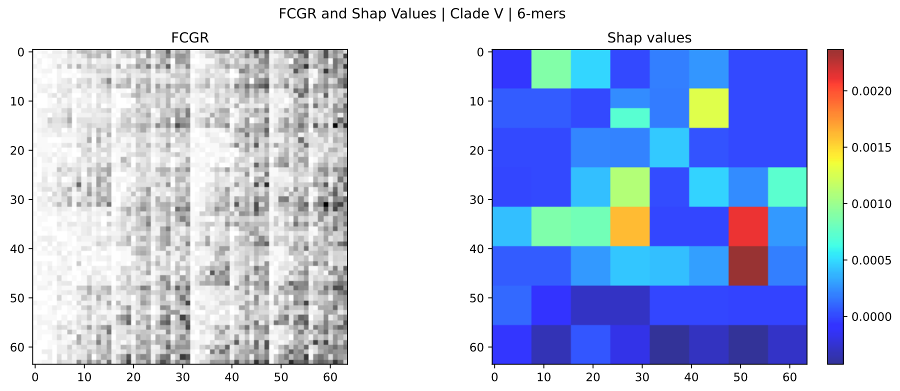

Figure 3. FCGR image (left) and Shap Values (right) of the centroid FCGR for the clade V ($k = 6$). The FCGR image is obtained rescaling the frequencies in the FCGR to a gray-scale range of 8 bits ([0,255]), an inversion of colors is performed to visualize higher values as black squares and lower values as white. Shap Values represent the importance of the features in the FCGR, the higher the value (red) the more important is the feature. Each feature (pixel) in the FCGR corresponds to a k-mer.

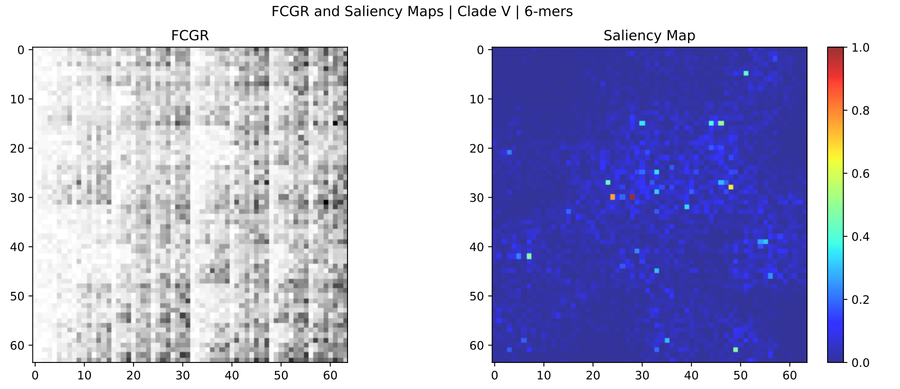

Figure 4. FCGR image (left) and Saliency Map (right) of the centroid FCGR for the clade V ($k = 6$). The FCGR image is obtained rescaling the frequencies in the FCGR to a gray-scale range of 8 bits ([0,255]), an inversion of colors is performed to visualize higher values as black squares and lower values as white. Saliency Map represent the importance of the features in the FCGR, the higher the value (red) the more important is the feature. Each feature (pixel) in the FCGR and Saliency Map corresponds to a k-mer.

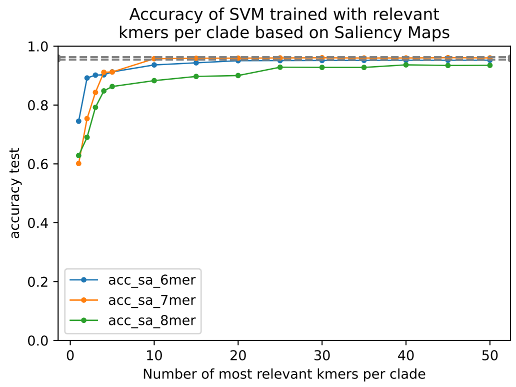

Figure 5. Accuracy of test set for SVM trained models using only the most N relevant k-mers for each clade ($N \in \{1, 2, 3, 4, 5, 10, 15, 20, 25, 30, 35, 40, 45, 50\}$). The relevant k-mers are selected using Saliency Maps on the centroid of the correctly classified FCGR for each clade and model. The same train and test datasets used for the trained CNNs are used for the SVM. The SVM trained with 20 most relevant k-mers identified by Saliency Map, for $k \in \{6, 7\}$ achieves an accuracy in the test set ($\approx$ 96%) that is in the range of the minimum and maximum accuracies (see Table 3) obtained by our trained CNNs (the gray dashed band represents the minimum and maximum accuracy for the trained CNNs).

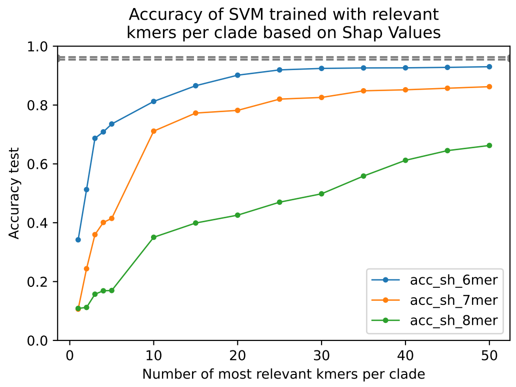

Figure 6. Accuracy of test set for SVM trained model using only the most N relevant k-mers for each clade ($N \in \{1, 2, 3, 4, 5, 10, 15, 20, 25, 30, 35, 40, 45, 50\}$). The relevant k-mers are selected using Shap Values on the centroid of the correctly classified FCGR for each clade and model. The same train and test datasets used for the trained CNNs are used for the SVM. The SVM trained with the 30 most relevant (or more) 6-mers identified by Shap Values, achieves the closest accuracy (92, 44%) to the ones obtained by our trained models (see Table 3). When k increases, the accuracy always decreases (for the same number of relevant k-mers), which can be explained since when k increases the total number of possible k-mers increases exponentially.

matches for $k = 6$, 51 for $k = 7$, and 11 for $k = 8$). On the other hand, the ones chosen by Shap Values barely match with the mutation (3 for $k = 6$), suggesting that Saliency Maps could provide a richer explainability of the model from a biological perspective.

Discussion

In this work we have shown that FCGR can be used to classify DNA sequences. Most notably, we have used FCGR to assign SARS-CoV-2

k-mer	Saliency Maps	Shap Values
6	46	3
7	51	0
8	11	0

Table 7. Summary of matches between the 50 most relevant k-mers (from Saliency Maps and Shap Values) and the list of marker variants reported by GISAID for each clade. The k-mers obtained by Saliency Maps are able to match several mutations and the matches decrease when k increases, but the ones from Shap Values only reported 3 matches, for $k = 6$.

genome sequences to its GISAID strain by running a CNN on 191, 456 genome sequences (80% training set, 10% validation set, and 10% test set). In particular, the 8-th order FCGR achieved a test accuracy of 96.22%. The majority of missclassified sequences are shared between two strongly related strains, GR and GRY (GR is a close ancestor of GRY).

We decided to exclude transfer learning from our experiments after trying this approach without success on 8-mers. For this trial we used pre-trained weights from the Imagenet dataset using Resnet50 architecture, where the backbone weights were frozen, and three dense layers were included at the top of it for the classification.

We have assessed the influence of the length k of the substrings (k-mers) used to build the FCGR, showing that values between 6 and 8 lead to very similar results, with less than 1% of difference in both accuracy and MCC on the same test set. However, when increasing the value of k, the training time for the model and the memory required to save the FCGRs increases exponentially. For $k = 6$ each epoch required 4:05 minutes and 6.6GB of memory, while for $k = 8$ it required 24:50 hour and 94.2GB. However, FCGRs show a fractal structures; this suggests that we might couple increasing k with using only a portion of the FCGR.

We compare our results with Covidex, a Random Forest based tool that classify sequences on GISAID clades based on k-mers frequencies. Under the same test set, our results show that our models outperform Covidex in all clades and reported metrics (accuracy, precision, recall and f1-score). Moreover, we found that the reported precision, recall and f1-score of Covidex are quite different for all clades but S and GV in our test set, exhibiting a decreasing in the f1-score metric up to 42.4%.

We have used Saliency Maps and Shap to identify relevant k-mers, looking for matches with the marker variants reported for each strain. Using the k-mers obtained by Saliency Map, we found 46, 51, and 11 matches for $k = 6, 7, 8$, respectively. While, for the k-mers identified by Shap, only 3 matches were found for $k = 6$. A possible direction for future works is to explore other existing methods (*e.g.* Lime [36], GradCAM [37], DeepLIFT [38]) that might be suitable in explaining the decisions of the model.

Classifying genome sequences introducing the assembly bias includes more factors to take care of, since any classification depends on the specific assembly pipeline that has been used. To lessen this possible problem, we should study a related problem, where we classify read samples instead of fully assembled genomes. This new problem is more complex, since different regions of the viral genome can have different coverage — hence impacting the frequencies — and reads needs to be cleaned from both errors and contamination artifacts (the latter might be attacked with specialized tools like KMC3 [39]).

We did not perform an extensive comparison of the running times since both tools classify a genome sequence in less than a second.

Potential implications

This paper shows how to couple Frequency Chaos Game Representation with a deep neural network that is especially suited to represent images, such as a CNN, to predict clade assignment. Since FCGR is a simple and intuitive representation of a set of k-mers, we expect this combination to find applications in several other problems that are currently attacked with approaches based on k-mers.

Methods

We use the k-th order FCGR representation for each sequence. In order to obtain this representation, we need to count the k-mers in each sequence and to put those frequencies in the FCGR based on the CGR encoding.

Before feeding the FCGR to the model, we rescale its elements to values between 0 and 1 for stability of the learning process. To do so, we divide each FCGR element-wise by the maximum value in the FCGR. It is worth mentioning that other preprocessing steps were taken into consideration but were ultimately excluded because found empirically worse.

Due to the huge amount of data available for most of the clades, we undersampled at most 20.000 sequences per clade to perform our experiments, nevertheless, for some clades only a portion of it was available (see table representativity). In order to overcome the unbalance in the undersampled dataset, we decided to use a weighted binary cross-entropy loss function (instead of oversampling the underrepresented classes), where the cost associated with a class c is inversely proportional to its representativity in the training set.

Model architecture

We choose a residual neural network, Resnet50 [40] as our CNN, adapted for k-th order FCGR, i.e. with input size equal to $(2^k \times 2^k \times 1)$, and output size equal to the number of clades: $|\mathcal{C}|$, with softmax activation function in the last layer and categorical crossentropy as loss function, since we want to assign only one clade to each DNA sequence.

Model evaluation

To assess the performance of our trained model, we perform a classification evaluation of the predictions and also a clustering evaluation for the embeddings in order to evaluate the class separability. The reported metrics are based on a Repeated 5-fold Cross Validation.

Classification metrics

We report global (accuracy and Matthews correlation coefficient) and class specific metrics (precision, recall, and F1-score) for the trained models.

Class specific metrics

Given a clade c, the correct predictions of the model can be compared to all the sequences with ground truth c (recall), and to all the sequences predicted by the model into the clade c (precision).

Formally, given a clade c, the positive class P consists of the set of genome sequences that are assigned to c, while all other genome sequences are the negative class N. Consequently, the true positive consist of the sequences that originate from the clade c and have been assigned to c, the false positive consist of the sequences that do not originate from the clade c and have been assigned to c, the false negative consist of the sequences that originate from the clade c and have not been assigned to c. The precision and recall are computed as follows:

$$precision = \frac{TP}{TP + FP}, \quad recall = \frac{TP}{TP + FN} \tag{3}$$

We also report the f1-score, defined as,

$$f1 - score = 2\frac{precision \times recall}{precision + recall} \tag{4}$$

Global model metrics

Given a classification problem on S samples and N classes, the corresponding confusion matrix $C = (c_{ij}), i, j \in [1, N]$ is a square matrix where each entry $c_{i,j}$ is the number of elements that belong to the true class i and were classified in the class j, and the sum of the entries in C is exactly S.

i. The **Accuracy** of the model is defined as the proportion of the corrected classified samples over the total number of samples, this value ranges between 0 and 1, where 0 means that all samples were erroneously classified, while a value of 1 means a perfect classification. It can be defined in terms of the entries of the confusion matrix as follows:

$$acc = \frac{\sum_{k=1}^{N} c_{kk}}{S} \tag{5}$$

ii. The **Matthews Correlation Coefficient** (MCC), proposed in [27] as a binary classification metric, was generalized to the multi-class case in 2004 [41], and it can be defined in terms of the confusion matrix as follows (see [42] for details):

$$MCC = \frac{cp \times S - \sum_{k=1}^{N} p_k \times t_k}{\sqrt{(S^2 - \sum_{k=1}^{N} p_k^2) \times (S^2 - \sum k = 1^N t_k^2)}} \tag{6}$$

where $cp = \sum_{k=1}^{N} c_{kk}$ is the total number of samples correctly predicted, $t_k = \sum_{i=1}^{N} c_{ik}$ is the number of times class k was truly occurred, and $p_k = \sum_{j=1}^{N} c_{kj}$ is the number of times class k was predicted. MCC lives in the range $[-1, 1]$, where 1 is perfect classification, −1 is the opposite, and 0 means that the confusion matrix is all zeros but for one single column, or when all entries are equal $c_{ij} = K \in \mathbb{N}$ [42].

Clustering measures

In order to assess the quality of the class separability given by the CNN, we evaluate the embeddings of the last layer (the one used to perform the classification) in the network with three clustering evaluation measures. These embeddings are the output from the final layer of the network for each FCGR.

i. **Silhouette Coefficient** [28] Given an embedding v belonging to a cluster A, the silhouette coefficient $s(v)$ of v compares the mean intra-cluster distance in A (a) with the mean nearest-cluster distance for v (b), that is, the closest cluster to v different from A.

$$s(v) = \frac{a - b}{\max\{a, b\}} \tag{7}$$

where $a = \frac{1}{|A|} \sum_{w \in A, w \neq v}^{|A|} d(v, w)$ and $b = \min_{B \neq A} \frac{1}{|B|} \sum_{w \in B}^{|B|, v \in A} d(v, w)$.
The value of $s(v)$ ranges between −1 (wrongly assigned) and 1 (perfect separability). For a cluster A, the mean silhouette coefficient of A is computed as the average of $s(v)$ over all embeddings $v \in A$.

ii. **Calinski-Harabasz Score** [29] Given a set of embeddings E of size n_E that has been clustered into k clusters, the Calinski-Harabasz Score s, also known as the Variance Ratio Criterion, is defined as the ratio of the between-clusters dispersion and the inter-cluster dispersion for all clusters (the dispersion of a group of n points is measured by the sum of the squared distances of the points from their centroid).

$$s = \frac{tr(B_k)}{tr(W_k)} \frac{n_E - k}{k - 1} \tag{8}$$

where $tr(B_k)$ is the trace of the between-cluster dispersion matrix and $tr(W_k)$ is the trace (the sum of all elements in the diagonal of W_k) of the within-cluster dispersion matrix, defined as follow:

$$W_k = \sum_{q=1}^{k} \sum_{v \in C_q} (v - c_q)(v - c_q)^T \tag{9}$$

$$B_k = \sum_{q=1}^{k} n_q (c_q - c_E)(c_q - c_E)^T \tag{10}$$

where C_q is the set of embeddings in the cluster q, c_q is the centroid of the cluster q, c_E is the centroid of E and $n_q = |C_q|$.
The higher the score s means that the clusters are dense and well separated.

iii. **Generalized Discrimination Value (GDV)** [30]
Given a set of N D−dimensional embeddings $\{x_1, \ldots, x_N\}$, with $x_n = (x_{n,1}, \ldots, x_{n,D})$ and a set of L classes $\{C_1, \ldots, C_L\}$, where each x_n is assigned to one of the L distinct classes. Consider their z−scored points $(s_1, \ldots, s_N)$, with $s_i = (s_{i,1}, \ldots, s_{i,D})$, where $s_{n,d} = \frac{1}{2} \frac{x_{n,d} - \mu_d}{\sigma_d}$. Here $\mu_d = \frac{1}{N} \sum_{n=1}^{N} x_{n,d}$ denotes the mean, and $\sigma_d = \sqrt{\frac{1}{N} \sum_{n=1}^{N} (x_{n,d} - \mu_d)^2}$ the standard deviation of dimension d. Using the re-scaled data points $s_n = (s_{n,1}, \ldots, s_{n,D})$, the Generalized Discrimination Value Δ is calculated from the mean intra−class and inter−class distances as follows:

$$\Delta = \frac{1}{\sqrt{D}} \left[\frac{1}{L} \sum_{l=1}^{L} d_{intra}(C_l) - \frac{2}{L(L-1)} \sum_{l=1}^{L-1} \sum_{m=l+1}^{L} d_{inter}(C_l, C_m) \right] \tag{11}$$

where the mean intra−class for each class C_l is defined as

$$d_{intra}(C_l) = \frac{2}{N_l(N_l - 1)} \sum_{i=1}^{N_l - 1} \sum_{j=i+1}^{N_l} d(s_i^{(l)}, s_j^{(l)}) \tag{12}$$

and the mean inter−class for each pair of classes C_l and C_m is defined as follows,

$$d_{inter}(C_l, C_m) = \frac{1}{\sqrt{D}} \left[\frac{1}{N_l N_m} \sum_{i=1}^{N_l} \sum_{j=1}^{N_m} d(s_i^{(l)}, s_j^{(l)}) \right] \tag{13}$$

here N_k correspond to the number of points in class k, and $s_i^{(k)}$ is the ith point of class k. The quantity $d(a, b)$ is the distance between a and b, for our case, we considered the Euclidean distance. The value Δ range between −1 (perfect separability) and 0 (wrongly assigned),

Feature importance

After the model is trained, we can perform feature importance methods (also known as pixel attribution in case of images) to analyze the impact of each element of the FCGR in our prediction. We selected Saliency Maps [31] and Shap Values [32]. Saliency Maps calculate the gradient of the loss function for a specific desired class with respect to the input (FCGR) elements, the gradients are rescaled to $[0,1]$, where elements with values closer to 1 represent the more influential features for the input FCGR over the predicted class. Shap (Shapley Additive Explanations) Values is a game theoretic approach to explain the output of any machine learning model. It aims to explain the influence of each feature compared to the average model output over the dataset the model was trained on, it outputs positive and negative values, where positive values push the prediction higher, and negative values push the prediction lower. Using the most relevant features from both methods over the FCGR, we aim to identify the most relevant k-mers for the classification of each clade.

Using these methods we aim to analyze the most relevant k-mers for the classification of each clade in the trained models.

Availability of source code and requirements

- Project name: Classification of SARS-CoV-2 genome sequence with CGR and CNN
- Project home page: https://github.com/AlgoLab/CouGaR-g
- Operating system(s): e.g. Platform independent
- Programming language: Python 3.10.5
- Other requirements: Python 3.10+, tensorflow 2.10.0, scikit-learn 1.1.2, tqdm 4.63.0, pandas 1.5.0, biopython 1.79, Pillow 9.0.1, matplotlib 3.5.1, shap 0.41.0, opencv-python 4.6.0.66
- License: GNU GPL

Availability of supporting data and materials

The list of FASTA sequences and metadata can be downloaded from https://www.gisaid.org/ after creating an account and accepting the *Terms of Use*. The data used in this study was downloaded on April 04, 2022. Trained models and results of our experiments can be downloaded from https://doi.org/10.5281/zenodo.7185290

A web app version of CouGaR-g with all the trained models is available online at https://huggingface.co/spaces/BIASLab/sars-cov-2-classification-fcgr

Declarations

List of abbreviations

- CNN: Convolutional Neural Networks
- CGR: Chaos Game Representation
- ENA: European Nucleotide Archive
- FCGR: Frequency matrix of Chaos Game Representation
- GDV: Generalized Discrimination Value
- SVM: Support Vector Machine

Consent for publication

Not applicable.

Competing Interests

The authors declare that they have no competing interests.

Funding

This project has received funding from the European Union's Horizon 2020 Innovative Training Networks programme under the Marie Skłodowska-Curie grant agreement No. 956229.

This project has received funding from the European Union's Horizon 2020 Research and Innovation Staff Exchange programme under the Marie Skłodowska-Curie grant agreement No. 872539.

Author's Contributions

JAC and SA wrote the code, prepared the data and performed the experiments. JAC, SA, SC, PB and GDV devised the methods and analyzed the results. JAC, SC and GDV designed the experiments. All authors contributed to finalizing of the manuscript.

Acknowledgements

The authors would like to thank Yuri Pirola, Raffaella Rizzi, Luca Denti, Murray Patterson, and Sarwan Ali for many useful discussions on the topic.

References

1. Khare S, Gurry C, Freitas L, Schultz MB, Bach G, Diallo A, et al. GISAID's Role in Pandemic Response. China CDC Weekly 2021;3(49):1049–1051. https://weekly.chinacdc.cn//article/id/21792cdf-a54a-4a11-b6fe-68d50f817d91.
2. Hadfield J, Megill C, Bell SM, Huddleston J, Potter B, Callender C, et al. Nextstrain: real-time tracking of pathogen evolution. Bioinformatics 2018 Dec;34(23):4121–4123. https://academic.oup.com/bioinformatics/article/34/23/4121/5001388, publisher: Oxford Academic.
3. Chapter 15 - Immunodeficiency. In: Mak TW, Saunders ME, Jett BD, editors. Primer to the Immune Response (Second Edition) Boston: Academic Cell; 2014.p. 377–421. https://www.sciencedirect.com/science/article/pii/B9780123852458000157.
4. Ali S, Bello B, Chourasia P, Punathil RT, Zhou Y, Patterson M. PWM2Vec: An Efficient Embedding Approach for Viral Host Specification from Coronavirus Spike Sequences. arXiv:220102273 [cs, q-bio] 2022 Jan;http://arxiv.org/abs/2201.02273, arXiv: 2201.02273.
5. Ali S, Patterson M. Spike2Vec: An Efficient and Scalable Embedding Approach for COVID-19 Spike Sequences. In: 2021 IEEE International Conference on Big Data (Big Data); 2021. p. 1533–1540.
6. Ali S, Sahoo B, Ullah N, Zelikovskiy A, Patterson M, Khan I. A k-mer Based Approach for SARS-CoV-2 Variant Identification. In: Wei Y, Li M, Skums P, Cai Z, editors. Bioinformatics Research and Applications, vol. 13064 Cham: Springer International Publishing; 2021.p. 153–164. https://link.springer.com/10.1007/978-3-030-91415-8_14, series Title: Lecture Notes in Computer Science.
7. Jeffrey HJ. Chaos game representation of gene structure. Nucleic Acids Res 1990 Apr;18(8):2163–2170.
8. Almeida JS, Carriço JA, Maretzek A, Noble PA, Fletcher M. Analysis of genomic sequences by Chaos Game Representation. Bioinformatics 2001 May;17(5):429–437.
9. Deschavanne PJ, Giron A, Vilain J, Fagot G, Fertil B. Genomic signature: characterization and classification of species assessed by chaos game representation of sequences. Mol Biol Evol 1999 Oct;16(10):1391–1399.
10. Wang Y, Hill K, Singh S, Kari L. The spectrum of genomic signatures: from dinucleotides to chaos game representation. Gene

2005;346:173–185. https://www.sciencedirect.com/science/article/pii/S0378111904006481.
11. Löchel HF, Eger D, Sperlea T, Heider D. Deep learning on chaos game representation for proteins. Bioinformatics 2019 06;36(1):272–279. https://doi.org/10.1093/bioinformatics/btz493.
12. Dick K, Green JR. Chaos Game Representations amp; Deep Learning for Proteome-Wide Protein Prediction. In: 2020 IEEE 20th International Conference on Bioinformatics and Bioengineering (BIBE); 2020. p. 115–121.
13. Ren Y, Chakraborty T, Doijad S, Falgenhauer L, Falgenhauer J, Goesmann A, et al. Prediction of antimicrobial resistance based on whole-genome sequencing and machine learning. Bioinformatics 2021 10;38(2):325–334. https://doi.org/10.1093/bioinformatics/btab681.
14. Millán Arias P, Alipour F, Hill KA, Kari L. DeLUCS: Deep learning for unsupervised clustering of DNA sequences. PLOS ONE 2022 01;17(1):1–25. https://doi.org/10.1371/journal.pone.0261531.
15. Löchel HF, Heider D. Chaos game representation and its applications in bioinformatics. Computational and Structural Biotechnology Journal 2021;19:6263–6271.
16. Singer J, Gifford R, Cotten M, Robertson D. CoV-GLUE: a web application for tracking SARS-CoV-2 genomic variation; 2020.
17. Randhawa GS, Soltysiak MP, El Roz H, de Souza CP, Hill KA, Kari L. Machine learning using intrinsic genomic signatures for rapid classification of novel pathogens: COVID-19 case study. Plos one 2020;15(4):e0232391.
18. Sengupta DC, Hill MD, Benton KR, Banerjee HN. Similarity Studies of Corona Viruses through Chaos Game Representation. Comput Mol Biosci 2020 Sep;10(3):61–72.
19. Touati R, Haddad-Boubaker S, Ferchichi I, Messaoudi I, Ouesleti AE, Triki H, et al. Comparative genomic signature representations of the emerging COVID-19 coronavirus and other coronaviruses: High identity and possible recombination between Bat and Pangolin coronaviruses. Genomics 2020;112(6):4189–4202. https://www.sciencedirect.com/science/article/pii/S0888754320306364.
20. LeCun Y, Boser B, Denker JS, Henderson D, Howard RE, Hubbard W, et al. Backpropagation applied to handwritten zip code recognition. Neural computation 1989;1(4):541–551.
21. Lecun Y, Haffner P, Bottou L, Bengio Y. Object Recognition with Gradient-Based Learning. In: Contour and Grouping in Computer Vision Springer; 1999. .
22. Krizhevsky A, Sutskever I, Hinton GE. Imagenet classification with deep convolutional neural networks. Advances in neural information processing systems 2012;25.
23. Rizzo R, Fiannaca A, La Rosa M, Urso A. Classification Experiments of DNA Sequences by Using a Deep Neural Network and Chaos Game Representation. In: Proceedings of the 17th International Conference on Computer Systems and Technologies 2016 CompSysTech '16, New York, NY, USA: Association for Computing Machinery; 2016. p. 222–228. https://doi.org/10.1145/2983468.2983489.
24. Safoury S, Hussein W. Enriched DNA Strands Classification Using CGR Images and Convolutional Neural Network. In: Proceedings of the 2019 8th International Conference on Bioinformatics and Biomedical Science ICBBS 2019, New York, NY, USA: Association for Computing Machinery; 2019. p. 87–92. https://doi.org/10.1145/3369166.3369176.
25. Cacciabue M, Aguilera P, Gismondi MI, Taboga O. Covidex: An ultrafast and accurate tool for SARS-CoV-2 subtyping. Infection, Genetics and Evolution 2022;99:105261.
26. He K, Zhang X, Ren S, Sun J. Deep residual learning for image recognition. In: Proceedings of the IEEE conference on computer vision and pattern recognition; 2016. p. 770–778.
27. Matthews BW. Comparison of the predicted and observed secondary structure of T4 phage lysozyme. Biochimica et Biophysica Acta (BBA)-Protein Structure 1975;405(2):442–451.
28. Rousseeuw PJ. Silhouettes: A graphical aid to the interpretation and validation of cluster analysis. Journal of Computational and Applied Mathematics 1987;20:53–65. https://www.sciencedirect.com/science/article/pii/0377042787901257.
29. Caliński T, Harabasz J. A dendrite method for cluster analysis. Communications in Statistics-theory and Methods 1974;3(1):1–27.
30. Schilling A, Maier A, Gerum R, Metzner C, Krauss P. Quantifying the separability of data classes in neural networks. Neural Networks 2021;139:278–293. https://www.sciencedirect.com/science/article/pii/S0893608021001234.
31. Simonyan K, Vedaldi A, Zisserman A. Deep inside convolutional networks: Visualising image classification models and saliency maps. In: In Workshop at International Conference on Learning Representations Citeseer; 2014. .
32. Lundberg SM, Lee SI. A Unified Approach to Interpreting Model Predictions. In: Guyon I, Luxburg UV, Bengio S, Wallach H, Fergus R, Vishwanathan S, et al., editors. Advances in Neural Information Processing Systems 30 Curran Associates, Inc.; 2017.p. 4765–4774. http://papers.nips.cc/paper/7062-a-unified-approach-to-interpreting-model-predictions.pdf.
33. Abadi M, Agarwal A, Barham P, Brevdo E, Chen Z, Citro C, et al., TensorFlow: Large-Scale Machine Learning on Heterogeneous Systems; 2015. https://www.tensorflow.org/, software available from tensorflow.org.
34. Pedregosa F, Varoquaux G, Gramfort A, Michel V, Thirion B, Grisel O, et al. Scikit-learn: Machine learning in Python. Journal of machine learning research 2011;12(Oct):2825–2830.
35. Kingma DP, Ba J, Adam: A Method for Stochastic Optimization; 2017.
36. Ribeiro MT, Singh S, Guestrin C. " Why should i trust you?" Explaining the predictions of any classifier. In: Proceedings of the 22nd ACM SIGKDD international conference on knowledge discovery and data mining; 2016. p. 1135–1144.
37. Selvaraju RR, Das A, Vedantam R, Cogswell M, Parikh D, Batra D. Grad-CAM: Why did you say that? Visual Explanations from Deep Networks via Gradient-based Localization. CoRR 2016;abs/1610.02391. http://arxiv.org/abs/1610.02391.
38. Shrikumar A, Greenside P, Kundaje A. Learning important features through propagating activation differences. In: International conference on machine learning PMLR; 2017. p. 3145–3153.
39. Kokot M, Długosz M, Deorowicz S. KMC 3: counting and manipulating k-mer statistics. Bioinformatics 2017;33(17):2759–2761.
40. He K, Zhang X, Ren S, Sun J. Deep Residual Learning for Image Recognition. CoRR 2015;abs/1512.03385. http://arxiv.org/abs/1512.03385.
41. Gorodkin J. Comparing two K-category assignments by a K-category correlation coefficient. Computational biology and chemistry 2004;28(5-6):367–374.
42. Jurman G, Furlanello C. A unifying view for performance measures in multi-class prediction. arXiv preprint arXiv:10082908 2010;.

Personal Cover Click here to access/download;Personal Cover;cover.odt

UNIVERSITÀ DEGLI STUDI DI MILANO BICOCCA
DIPARTIMENTO DI INFORMATICA, SISTEMISTICA E COMUNICAZIONE
Viale Sarca, 336
20126 Milano

Gianluca Della Vedova

gianluca.dellavedova@unimib.it
https://www.unimib.it/gianluca-della-vedova

Milan, May 4th, 2022

Subject: submission to Gigascience

Dear Dr. Scott Edmunds,

Our group at the University of Milano-Bicocca has developed a new deep learning method for identifying the SARS-CoV-2 clade to which a genome sequence belongs, and we have prepared a manuscript, titled “Accurate and Fast Clade Assignment via Deep Learning and Frequency Chaos Game Representation”, for possible publication in Gigascience.

A fundamental technical contribution of our paper is the investigation of the Chaos Game Representation suitability for encoding SARS-CoV-2 genomes. The final result is a tool (CouGaR-g) that achieves over 95% accuracy for identifying the clade, over an extensive simulation study.

Since our initial submission a month ago, we have compared our tool with Covidex, showing that we consistently obtain better precision and recall than Covidex (Tables 4 and 5). Moreover, we have completely revised the paper, including writing new Data Description and the Potential Implications sections, and we have provided our tool as a web application (https://huggingface.co/spaces/BIASLab/sars-cov-2-classification-fcgr) for easier testing.

On behalf of all of my co-authors, I remain at your disposal for any further inquiries, and we look forward to hearing from you soon.

Sincerely,

Dr. Gianluca Della Vedova
