# Supplementary material for: Accurate and fast clade assignment via deep learning and frequency chaos game representation
Source: Gigascience. 2022 Dec 28;12:giac119. doi: 10.1093/gigascience/giac119 (PMC9795481; doi:10.1093/gigascience/giac119)
Supplement: giac119_Response_to_Reviewer_Comments_Revision_1 [file giac119_response_to_reviewer_comments_revision_1.pdf]

Author's Response To Reviewer Comments

Close

We corrected the comments reported in the manuscript

Close
