# Supplementary material for: Accurate and fast clade assignment via deep learning and frequency chaos game representation
Source: Gigascience. 2022 Dec 28;12:giac119. doi: 10.1093/gigascience/giac119 (PMC9795481; doi:10.1093/gigascience/giac119)
Supplement: giac119_Reviewer_1_Report_Original_Submission — Dominik Heider -- 6/1/2022 Reviewed [file giac119_reviewer_1_report_original_submission.pdf]

Reviewer Report

Title: Accurate and Fast Clade Assignment via Deep Learning and Frequency Chaos Game Representation

Version: Original Submission **Date:** 6/1/2022

Reviewer name: Dominik Heider

Reviewer Comments to Author:

The paper is well written, and the objectives are clear. The study is a very nice application of CGR in bioinformatics and shows the excellent performance of CGR-encoded data in combination with deep learning. I have a few things that should be addressed in a minor revision:
1) Some very important studies have not been addressed in the related work part, e.g., in Touati et al. (pubmed:32645523) and Sengupta et al. (pubmed:32953249), the authors compared SARS-CoV2 with other coronaviruses based on CGR, or we (pubmed:34613360) used CGR in combination with deep learning for resistance predictions in E. coli.
2) To me, it is unclear how accuracy was used in the model. Is it one class (i.e., clade) versus all others? If yes, accuracy might be misleading because of the high class imbalance. In such high class imbalances, MCC has been shown to be more suitable.
3) "The undersampled dataset was randomly split into train...". Why did you undersample? To balance the data, which would make sense to use accuracy as a metric but discard a lot of valuable data. What about oversampling?
4) Comparison with other tools: I wonder whether the good performance of your model is the result of deep learning or the CGR encoding. Please also provide the results for another ML model (besides SVM, e.g., random forests) to compare to, e.g., Covidex.

Methods

Are the methods appropriate to the aims of the study, are they well described, and are necessary controls included? Choose an item.

Conclusions

Are the conclusions adequately supported by the data shown? Choose an item.

Reporting Standards

Does the manuscript adhere to the journal's guidelines on [minimum standards of reporting?](minimum standards of reporting) Choose an item.

Choose an item.

Statistics

Are you able to assess all statistics in the manuscript, including the appropriateness of statistical tests used? Choose an item.

Quality of Written English

Please indicate the quality of language in the manuscript: Choose an item.

Declaration of Competing Interests

Please complete a declaration of competing interests, considering the following questions:

- Have you in the past five years received reimbursements, fees, funding, or salary from an organisation that may in any way gain or lose financially from the publication of this manuscript, either now or in the future?
- Do you hold any stocks or shares in an organisation that may in any way gain or lose financially from the publication of this manuscript, either now or in the future?
- Do you hold or are you currently applying for any patents relating to the content of the manuscript?
- Have you received reimbursements, fees, funding, or salary from an organization that holds or has applied for patents relating to the content of the manuscript?
- Do you have any other financial competing interests?
- Do you have any non-financial competing interests in relation to this paper?

If you can answer no to all of the above, write 'I declare that I have no competing interests' below. If your reply is yes to any, please give details below.

I declare that I have no competing interests

I agree to the open peer review policy of the journal. I understand that my name will be included on my report to the authors and, if the manuscript is accepted for publication, my named report including any attachments I upload will be posted on the website along with the authors' responses. I agree for my report to be made available under an Open Access Creative Commons CC-BY license (http://creativecommons.org/licenses/by/4.0/). I understand that any comments which I do not wish to be included in my named report can be included as confidential comments to the editors, which will not be published.

Choose an item.

To further support our reviewers, we have joined with Publons, where you can gain additional credit to further highlight your hard work (see: https://publons.com/journal/530/gigascience). On publication of this paper, your review will be automatically added to Publons, you can then choose whether or not to claim your Publons credit. I understand this statement.

Yes Choose an item.
