# Supplementary material for: Accurate and fast clade assignment via deep learning and frequency chaos game representation
Source: Gigascience. 2022 Dec 28;12:giac119. doi: 10.1093/gigascience/giac119 (PMC9795481; doi:10.1093/gigascience/giac119)
Supplement: giac119_Reviewer_2_Report_Original_Submission — Riccardo Rizzo -- 7/19/2022 Reviewed [file giac119_reviewer_2_report_original_submission.pdf]

Reviewer Report

Title: Accurate and Fast Clade Assignment via Deep Learning and Frequency Chaos Game Representation

Version: Original Submission **Date:** 7/19/2022

Reviewer name: Riccardo Rizzo

Reviewer Comments to Author:

The authors propose a classification experiment based on Frequency Chaos Game Representation and deep learning. They used the outstanding performances of a ResNet network as an image classification tool and the FCGR method that represent a genome sequence as an image.
The work seems good, although some major points should be clarified.
First, whether the performance index values came from a 5-fold validation procedure (5 because they said the split was 80-10-10) or a one-shot experiment is unclear.
Second, the part that involves the frequent k-mers and the SVM should be better explained. The authors should clarify what the meaning of this comparison is.
Another point to clarify is the quality of the sequences used; the authors worked on complete sequences, but, as far as I know, in the real world virus sequences are noisy data, and authors should discuss this point.
Minor points:
- Authors said that a sequence is a string $s \in \{A, C, G, T, N\}^*$, so they should explain the procedure used in Definition 2, where only 4 symbols seem to be used. If they discard the N, or consider 4 k-mers (consider that N means "any symbol") they should say it clearly.
- Figure 1 and 2 report two different quantities but say the same thing; maybe one of them can be omitted.
- Authors should add some details about the training time of the network.
A final suggestion: probably it will be interesting to use the same deep network with transfer learning (the whole network or just the first sections) to evaluate the gain with ad-hoc training and the different training time.

Methods

Are the methods appropriate to the aims of the study, are they well described, and are necessary controls included? Choose an item.

Conclusions

Are the conclusions adequately supported by the data shown? Choose an item.

Reporting Standards

Does the manuscript adhere to the journal’s guidelines on [minimum standards of reporting?] Choose an item.

Choose an item.

Statistics

Are you able to assess all statistics in the manuscript, including the appropriateness of statistical tests used? Choose an item.

Quality of Written English

Please indicate the quality of language in the manuscript: Choose an item.

Declaration of Competing Interests

Please complete a declaration of competing interests, considering the following questions:

- Have you in the past five years received reimbursements, fees, funding, or salary from an organisation that may in any way gain or lose financially from the publication of this manuscript, either now or in the future?
- Do you hold any stocks or shares in an organisation that may in any way gain or lose financially from the publication of this manuscript, either now or in the future?
- Do you hold or are you currently applying for any patents relating to the content of the manuscript?
- Have you received reimbursements, fees, funding, or salary from an organization that holds or has applied for patents relating to the content of the manuscript?
- Do you have any other financial competing interests?
- Do you have any non-financial competing interests in relation to this paper?

If you can answer no to all of the above, write 'I declare that I have no competing interests' below. If your reply is yes to any, please give details below.

I declare that I have no competing interests

I agree to the open peer review policy of the journal. I understand that my name will be included on my report to the authors and, if the manuscript is accepted for publication, my named report including any attachments I upload will be posted on the website along with the authors' responses. I agree for my report to be made available under an Open Access Creative Commons CC-BY license (http://creativecommons.org/licenses/by/4.0/). I understand that any comments which I do not wish to be included in my named report can be included as confidential comments to the editors, which will not be published.

Choose an item.

To further support our reviewers, we have joined with Publons, where you can gain additional credit to further highlight your hard work (see: https://publons.com/journal/530/gigascience). On publication of this paper, your review will be automatically added to Publons, you can then choose whether or not to claim your Publons credit. I understand this statement.

Yes Choose an item.
